# Role of PI3K/AKT pathway in cancer: the framework of malignant behavior

**DOI:** 10.1007/s11033-020-05435-1

**Published:** 2020-04-24

**Authors:** Ningni Jiang, Qijie Dai, Xiaorui Su, Jianjiang Fu, Xuancheng Feng, Juan Peng

**Affiliations:** 1 Department of Pathology, The Third Affiliated Hospital of Guangzhou Medical University, 63 Duobao Road, Guangzhou, 510150 China; 2 The Third Clinical School of Guangzhou Medical University, Guangzhou, 510150 China; 3 Key Laboratory of Reproduction and Genetics of Guangdong Higher Education Institutes, Guangzhou, 510150 China; 4 Department of Microbiology and Immunology, Wake Forest School of Medicine, Winston-Salem, NC 27157 USA

**Keywords:** PI3K, AKT, PTEN, Cancer, Targeted therapy

## Abstract

Given that the PI3K/AKT pathway has manifested its compelling influence on multiple cellular process, we further review the roles of hyperactivation of PI3K/AKT pathway in various human cancers. We state the abnormalities of PI3K/AKT pathway in different cancers, which are closely related with tumorigenesis, proliferation, growth, apoptosis, invasion, metastasis, epithelial–mesenchymal transition, stem-like phenotype, immune microenvironment and drug resistance of cancer cells. In addition, we investigated the current clinical trials of inhibitors against PI3K/AKT pathway in cancers and found that the clinical efficacy of these inhibitors as monotherapy has so far been limited despite of the promising preclinical activity, which means combinations of targeted therapy may achieve better efficacies in cancers. In short, we hope to feature PI3K/AKT pathway in cancers to the clinic and bring the new promising to patients for targeted therapies.

## Background

Cancer is considered as the major cause of mortality in the worldwide. According to the global cancer statistics of the Global Cancer Observatory (GCO), there will be 18.1 million new cases and 9.6 million cancer deaths worldwide in 2018 (World Health Organization. Cancer. 2018; https://gco.iarc.fr/). The top 5 most prevalent cancers in the world are lung cancer (LC), breast cancer (BC), prostate cancer (PCa), colon cancer and gastric cancer (GC, Table 1). In China, LC and liver cancer were two of the top five causes of death leading to years of life lost (YLLs) in 2017 [1]. Environmental and genetic risk factors have been recognized as the two major risk factors resulting in various tumorigenesis and cancer progression. Recent decades have witnessed the molecular understanding of the mechanisms of numerous genetic factors in human cancer, such as phosphatidylinositol 3-kinase/protein kinase B (PI3K/AKT), P53, NF-kB, STAT3, COX-2 and c-Myc. Apparently, PI3K/AKT pathway has gradually gotten a major focus of attention as it plays a crucial role in regulating diverse cellular functions, including metabolism, growth, proliferation, survival, transcription and protein synthesis.

**Table 1:** Incidence, mortality and genetic alteration of PI3K/AKT pathway by cancer site (https://gco.iarc.fr/; https://www.cbioportal.org/)

System	Cancer	Incidence rate (%)	Mortality rate (%)	Subtype of cancer	Genetic alteration of PI3K/AKT pathway (%)					
					*PIK3CA*	*PIK3R1*	*PIK3R2*	*AKT1*	*AKT2*	*PTEN*
Brain and Central Nervous	Tumors	1.6	2.5	GBM	7	6	0.7	0.9	0.3	22
				MBM	2	0.3		0.3		1.3
Endocrine	TC	3.1	0.4	TC	1.8	0.3	0.5	0.5	0.5	2.3
				ATC	18	0	3			15
				PDTC	2	1	0			4
Respiratory	NPC	0.7	0.8		1.8					
	LC	11.6	18.4	NSCLC	17	1.8	1.6	2.1	3	6
				SCLC	3	2	1.5	0.5	1.5	8
Digestive	ESCA	3.2	5.3		24	2.7	1.6	3	1.6	7
	GC	5.7	8.2		17	4	2.5	1.4	2.8	11
	Colon cancer	6.1	5.8		21	4	4	2.2	3	9
	RC	3.9	3.2							
	CRC				22	5	2.2	1.8	1.5	8
	HCC	4.7	8.2		3	1.2	1.5	0.7	1.1	4
	GBC	1.2	1.7		10	0.8	0	1.5	1.5	2.3
	PC	2.5	4.5		2.3	0.7	1.2	2.2	3	1.9
Breast and female reproductive	BC	11.6	6.6		37	3	1.9	5	1.6	8
	OC	1.6	1.9		29	5	9	5	8	7
	CC	3.2	3.3		39	4	1.1	4	5	13
	EC	2.1	0.94		34	19	5	3	5	32
Genitourinary	PCa	7.1	3.8		6	4	1.9	2.5	1.3	18
	BLCA	3.0	2.1		24	3	1.1	3	2.5	6
	KC	2.2	1.8		2.8	0.4	0.3	0.5	0.6	4
	Te Ca	0.39	0.1		3	1.3		0.7		0.7
Hematologic	HL	0.44	0.27							
	NHL	2.8	2.6		0.4	0.5	0.1	0.1	0.1	1.1
	MM	0.88	1.1							
	Leukemia	2.4	3.2		0.6	0.6	0.4	0.5	0.1	0.7
Bone and soft tissue	OS				1/59	1/59		1/59		7/59 [366]
	EWS				1.4	0.5				0.5
Skin	Melanoma	1.6	0.64		5	2	1.5	1.7	1.7	12

*BC* breast cancer, *BLCA* bladder cancer, *CRC* colorectal carcinoma, *EC* endometrial cancer, *ESCA* esophageal cancer, *EWS* Ewing's sarcoma, *GBM* glioblastoma, *GC* gastric cancer, *HCC* hepatocellular carcinoma, *HL* Hodgkin's lymphoma, *KC* kidney cancer, *LC* lung cancer, *MBM* medulloblastoma, *MM* multiple myeloma, *NHL* non-Hodgkin's lymphoma, *NSCLC* non-small cell lung cancer, *OC* ovarian cancer, *OS* osteosarcoma, *PC* pancreatic cancer, *PCa* prostate cancer, *SCLC* small cell lung cancer, *TC* thyroid cancers, *Te Ca* testicular cancer

The PI3Ks are a family of heterodimeric lipid kinases, which are grouped into class I, II, and III isoforms. Class IA subgroup of PI3Ks activated by receptor tyrosine kinases consist of a p110 catalytic subunit (p110α, *PIK3CA*; p110β, *PIK3CB*; p110δ, *PIK3CD*) and one of five p85-like regulatory subunits (p85α, p55α, p50α, *PIK3R1*; p85β, *PIK3R2*; p55γ, *PIK3R3*). Class IB subgroup of PI3Ks activated by G protein-coupled receptors consist of the catalytic subunit (p110γ, *PIK3CG*) and regulatory subunits (p101, *PIK3R5*; p87, *PIK3R6*). Class II PI3Ks comprises PI3K-C2α (*PIK3C2A*), β (*PIK3C2B*) and γ (*PIK3C2G*). And the single class III PI3K is hVPS34 (*PIK3C3*). When PI3K is activated by a variety of upstream cell-surface receptors, including growth factor, antigen, costimulatory, cytokine, chemokine, and Toll-like receptors (TLRs), class I PI3Ks catalyzes the conversion of phosphatidylinositol 4,5-bisphosphate (PI(4,5)P_2_) with phosphorylation at the D3 position of the inositol ring to the second messenger phosphatidylinositol 3,4,5-triphosphate (PIP_3_). Two PIP_3_-binding Pleckstrin homology (PH) domain-containing proteins linked to PI3K activity in all cells, including B cells, are the serine/threonine kinases AKT and phosphoinositide-dependent kinase-1 (PDK-1) [2–5].

AKT is an evolutionarily conserved serine protein kinase from the protein kinase AGC subfamily, which is composed of three conservative structure domains, including N-terminal PH domain, a short C-terminal tail containing a regulatory hydrophobic motif (HM) and a linker region with a central kinase catalytic domain [6]. AKT contains three highly conserved homologous subtypes, AKT1/PKBα (*AKT1*), AKT2/PKBβ (*AKT2*) and AKT3/PKBγ (*AKT3*). On the cell membrane, AKT is recruited via its PH domain ascribing to the accumulation of PI(3,4,5)P_3_ and PI(3,4)P_2_ (less extent), and plays a catalytic role by activating two regulatory sites, including a threonine phosphorylated by PDK1 at Thr308(AKT1), Thr309(AKT2), Thr305(AKT3) and a serine phosphorylated by the mammalian Target of Rapamycin (mTOR) Complex mTORC2 at Ser473(AKT1), Ser474(AKT2), Ser472(AKT3) respectively as well as specifically [7, 8]. Massive researches have shown that AKT regulates vital downstream effector molecules, such as FOXO, mTOR, GSK3b, and many other effectors via phosphorylation cascade reaction, which is modulated by lipid and protein phosphatases, to control cell growth, proliferation, survival, genome stability, glucose metabolism, and neovascularization [9–12]. However, the activities of these phosphatases are frequently lost or inactivated evidently in human cancer, followed by the result of AKT hyperactivation.

When talking about PI3K/AKT pathway, we have to mention phosphatase and tensin homolog deleted on chromosome 10 (PTEN), the primary negative regulator of the PI3K/AKT pathway. As a lipid phosphatase, PTEN directly suppresses the activation of PI3K/AKT pathway via converting the PIP_3_ generated by PI3K back to PIP_2_. The p85α regulatory subunit has a dual effect on the p110α catalytic subunit, since p85α inhibits the activity of p110α while it plays an important role in the stability of p110α. In addition, the p85α regulatory subunit has been proven to directly bind PTEN and enhance its activity to promote the conversion of PIP3 to PIP_2_ [13, 14]. Indeed, the abnormality of PTEN have been validated in diverse cancers, even directly related with carcinogenesis in some cancers.

Following the emerging alterations of PI3K/AKT pathway genes have been widely reported in cancers recently, the inhibitors of PI3K/AKT pathway have brought a new era for targeted therapy of cancer. Since the first approval of idelalisib (CAL-101) validated the druggability of the PI3K pathway, more and more PI3K inhibitors have been created. They are generally divided into pan-PI3K (targeting all four isoforms of class I PI3K), isoform-selective (targeting single isoform of class I PI3K) and dual inhibitors (highlighted by dual PI3K/mTOR inhibitors). Comparatively, the number of AKT inhibitors which have been explored in clinical trials is less than that of PI3K inhibitors. AKT inhibitors mainly include two separate classes: Allosteric inhibitors and ATP-competitive inhibitors. The formers prevent localisation of AKT by PH domain to the plasma membrane, thereby blocking AKT phosphorylation and activation. The latters targeting the phosphorylated conformation of AKT include first generation and second generation inhibitors [15, 16]. These PI3K/AKT inhibitors have shown their various aptitude for anticancer in preclinical experiments or clinical trials, even druggable value for the anticancer treatment.

In this review, we present the comprehensive work of PI3K/AKT pathway with a new perspective in various cancer sites, in which elevated PI3K/AKT pathway is considered as a hallmark. Firstly, we state the abnormalities of PI3K/AKT pathway and summarize the roles of PI3K/AKT in aberrant signaling cascades in human cancers. Furthermore, we list the involvement of the PI3K/AKT inhibitors in the clinical trials of targeted therapies in cancers. Meanwhile, we briefly provide preliminary findings in the context of resistance to targeted therapies. Finally, we discuss the confusion and the future of the PI3K/AKT pathway.

## Recent studies and results

### Profiling the PI3K/AKT pathway in the brain and central nervous system tumors

Considering that the incidence and mortality of the brain and central nervous system tumors is 1.6% and 2.5% respectively in the worldwide (https://gco.iarc.fr/, Table 1), particularly the most common primary malignant tumor, glioblastoma multiforme (GBM), contributes to the poor prognosis partly for its tolerance of radiation therapy, hyper-activation of PI3K/AKT pathway in GBM caused by the mutations of *PIK3CA* or *PIK3R1* (18.3%) and other PI3K family genes (6.8%) has urged researchers to seek novel targeted treatments to control the disease [17–19]. Moreover, knockdown of *PIK3CA* or *PIK3R1* significantly inhibits cell viability, migration and invasion in GBM cells via hypo-activation of AKT and FAK [20]. In addition, overexpression of p110β is more frequently detected in a series of GBM cell lines than in the patient tumor samples. *PIK3CB* knockdown suppresses cell proliferation and induces caspase-dependent apoptosis in GBM in *vitro* and *vivo* instead of suppressing GBM cell migration [21–23]. Therefore, PI3K inhibitors have been seriously studied in GBM for decades and some have achieved significant success in treating GBM.

As a matter of fact that more than 50 PI3K inhibitors have been designed and produced for cancer treatment, but only a minority of them such as BKM120, XL147, XL765 and GDC-0084 have successfully entered into clinical trials for GBM treatment (https://clinicaltrials.gov, Table 2) [18]. Some p110α isoform-selective inhibitors, such as A66 or PIK-75, could effectively suppress the GBM cell growth, survival and migration in vitro [24], while inhibition of p110β by TGX-221 only arrests cell migration, and inhibition of p110δ by IC87114 or CAL-101 moderately blocks cell proliferation and migration [22, 25]. However, PI3K inhibitors including A66 and BEZ235 are observed to increase the expression of cancer stem cell (CSC) genes (SOX2, OCT4 and MSI1) in GBM CSC models, which exhibit therapy resistance [26].

**Table 2:** Clinical trial of PI3K Inhibitors in cancers (as of December 2019) (https://clinicaltrials.gov)

System	Cancer	Subunit	Inhibitors	Characteristic	Clinical trials	
					Phase	Gov identifier
Brain and central nervous	GBM	*Pan-*	BKM120	To assess the safety and the dose of the combination of INC280 and BKM120, as well as the anti-tumor activity of the combination, in patients with recurrent GBM with mutations or homozygous deletion of *PTEN* or PTEN negative by IHC	I/II	NCT01870726
			XL147	To measure what effect XL147 has on tumor tissue in subjects with recurrent GBM who are candidates for surgical resection	I	NCT01240460
		*Dual*	GDC-0084	To assess the safety, PK and Efficacy of GDC-0084 in newly-diagnosed GBM	II	NCT03522298
			XL765	To measure what effect XL765 has on tumor tissue in subjects with recurrent GBM who are candidates for surgical resection	I	NCT01240460
	MBM	*Dual*	LY3023414	To study how well LY3023414 works in treating patients with recurrent MBM2	II	NCT03213678
	UM	*IS*	BYL719	Phase Ib Trial of AEB071 in combination with BYL719 in patients with metastatic UM	I	NCT02273219
Endocrine	TC	*Pan-*	BKM120	Evaluating the efficacy and safety of BKM120 in the treatment of patients with advanced or metastatic differentiated TC	II	NCT01830504
		*Dual*	CUDC-907	To see if CUDC-907 will shrink tumors in people with advanced TC	II	NCT03002623
	PNETs	*IS*	BYL719	To study the safety and efficacy of BYL719 with Everolimus or BYL719 with Everolimus and Exemestane in advanced PNETs	I	NCT02077933
Respiratory	SCLC	*Pan-*	BKM120	Combine BKM120 with cisplatin and etoposide may kill more tumor cells	I	NCT02194049
	NSCLC	*Pan-*	BKM120	BKM120 and pemetrexed disodium may stop the growth of tumor cells by blocking some of the enzymes needed for cell growth. Giving BKM120, carboplatin, and pemetrexed disodium together may kill more tumor cells	I	NCT01723800
				The safety, tolerability and RP2D of the combination of gefitinib and BKM120 will be determined	I	NCT01570296
				Giving BKM120, gemcitabine hydrochloride, and cisplatin may be a better treatment for solid tumors	I	NCT01971489
				To determine the MTD/RP2D of BKM120 in combination with docetaxel. Subsequently the MTD/RP2D will be investigated in a Phase II randomized trial in patients with advanced or metastatic squamous NSCLC	I	NCT01911325
			GDC-0032	To explore the effects of GDC-0032 in treating patients with stage IV squamous cell lung cancer	II	NCT02785913
			GDC-0941	This is an open-label, multicenter, Phase Ib dose-escalation study to assess the safety, tolerability and PO of GDC-0941	I	NCT00974584
		*IS*	CAL-101	To determine the safety and effectiveness of the combination of pembrolizumab and CAL-101 in NSCLC patients who has stopped responding to immune therapy and see if adding CAL-101 to pembrolizumab will increase response rates vs. pembrolizumab alone	I/II	NCT03257722
			BYL719	To evaluate the overall response rate of NSCLC patients	II	NCT02276027
			AZD8186	To explore the efficacy of AZD8186 as monotherapy or in combination with abiraterone acetate or AZD2014 in patients with squamous NSCLC	I	NCT01884285
		*Dual*	PKI-587	Study of PD-0332991 in combination with PKI-587 for patients with advanced squamous cell lung solid tumors	I	NCT03065062
				To determine if PKI-587 given in combination with paclitaxel and carboplatin will work against unresectable NSCLC	I/II	NCT02920450
			LY3023414	To find a recommended dose level and schedule of dosing LY3023414 that can safely be taken by participants with advanced or metastatic cancer	I	NCT01655225
	NPC	*Pan-*	BKM120	To study the SE and BD of BKM120 in combination with cetuximab and how well it works in treating patients with recurrent or metastatic head and neck cancer	I/II	NCT01816984
	LSCC	*Pan-*	BKM120	To assess tolerability of the combining standard chemoradiotherapy with weekly cisplatin and BKM120 in high risk patients with locally advanced SCCHN	I	NCT02113878
Digestive	ESCC	*Pan-*	BKM120	BKM120 is currently tested in clinical trials, and it is used for patients with ESCC after failure of first line chemotherapy	II	NCT01806649
		*IS*	BYL719	During or after palliative first-line platinum-based chemotherapy, patients with ESCC will be screened for NGS-based molecular screening. The patients with the genetic alteration of PI3Ks will be treated with BYL719 and be observed its efficacy	II	NCT03292250
	GC	*Pan-*	BKM120	To determine the MTD and/or RP2D of a combination of imatinib and BKM120 in the treatment of 3rd line GIST patients	I	NCT01468688
		*IS*	GSK2636771	To evaluate the safety, PK and clinical activity of GSK2636771 administered in combination with Paclitaxel in advanced GC having alterations in PI3K pathway genes	I/II	NCT02615730
				To evaluate the ORR of patients targeted study agent(s) in patients with advanced refractory cancers	II	NCT02465060
			BYL719	To investigate the safety of BYL719 and AUY922 in patients with advanced GC, and to determine the MTD and/or RDE of both drugs in combination	I	NCT01613950
	CRC	*Pan-*	BKM120	To determine whether treatment with BKM120 demonstrates sufficient efficacy in patients with PI3K-activated tumors, such as CR, OC to warrant further study	II	NCT01833169
		*IS*	BYL719	To assess the safety and efficacy of LGX818 when combined with cetuximab or combined with cetuximab and BYL719 in patients with *BRAF* mutant metastatic CRC	I/II	NCT01719380
			TAK-117	To test if combining TAK-117 with canagliflozin will improve efficacy in the treatment of advanced solid tumors	I/II	NCT04073680
		*Dual*	DS-7423	To determine the MTD and measure the effects of DS-7423 on the patients with advanced CRC	I	NCT01364844
			LY3023414	To evaluate the safety of LY3039478 in combination with other anticancer agents including LY3023414 in participants with advanced or metastatic solid tumors	I	NCT02784795
	GIST	*Pan-*	BKM120	To determine the MTD and/or RP2D of a combination of imatinib and BKM120 in the treatment of 3rd line GIST patients	I	NCT01468688
		*IS*	BYL719	To determine the MTD and/or RP2D of a combination of imatinib and BYL719 in the treatment of 3rd line GIST patients	I	NCT01735968
	HCC	*Dual*	SF1126	To determine the MTD or MRD and the RP2D of SF1126 in combination with nivolumab in adult patients with advanced HCC	I	NCT03059147
		*IS*	GSK2636771	To evaluate the ORR of patients targeted study agent(s) in patients with advanced refractory cancers	II	NCT02465060
	PC	*Pan-*	BKM120	To investigate the safety, PK and PD of BKM120 plus GSK1120212 in advanced *RAS* or *BRAF* mutant PC patients	I	NCT01155453
				To investigate the safety, PK and PD of BKM120 plus MEK162 in advanced *RAS* or *BRAF* mutant PC patients	I	NCT01363232
		*IS*	GSK2636771	To evaluate the ORR of patients targeted study agent(s) in patients with advanced refractory cancers	II	NCT02465060
			BYL719	To see primarily if BYL719 is safe to be given to patients in combination with gemcitabine and nab-paclitaxel in locally advanced and metastatic PC	I	NCT02155088
		*Dual*	PKI-587	To study PD-0332991 in combination with PKI-587 for patients with advanced PC solid tumors	I	NCT03065062
			BEZ235	To study the safety, PK and PD of BEZ235 Plus MEK162 in advanced PC solid tumor patients	I	NCT01337765
			LY3023414	To evaluate the safety and efficacy of abemaciclib alone and in combination with other drugs including LY3023414 in participants with previously treated metastatic PDAC	II	NCT02981342
Reproductive	BC	*Pan-*	BKM120	Evaluating the clinical activity of BKM120 in patients with metastatic TNBC	II	NCT01629615
				Evaluating BKM120 in combination with trastuzumab and paclitaxel in HER2 + primary BC	II	NCT01816594
				Evaluating the safety profile/tolerability and preliminary anti-tumor effect of BKM120 and endocrine therapy combination and BEZ235 and endocrine therapy combination in postmenopausal patients with HR + MBC	I	NCT01248494
				To determine whether treatment with BKM120 plus letrozole led to an increase in pathologic clinical response and ORR compared to treatment with placebo plus letrozole in patients with BC	II	NCT01923168
				To assess the MTD and/or RP2Ds, safety and tolerability, the single and multiple dose PK profile and assess the preliminary antitumor activity of BYL719 and BKM120 in combination with tamoxifen plus goserelin acetate in premenopausal advanced HR + BC patients	I	NCT02058381
				BKM120 and anti-HER2 therapy may have a synergistic antitumor activity in preclinical model of HER2 + BC	I/II	NCT01589861
				To determine the MTD and /or RP2D and schedule for BKM120 given in combination with GSK1120212 in patients with selected, advanced solid tumors	I	NCT01155453
				Inhibition of PI3K by BKM120 may enhance apoptosis in ER + BC cells	I	NCT01339442
				To look for MTD, and also to see if the combination of BKM120 or BYL719 and olaparib is effective in treating BC	I	NCT01623349
				To explore the efficacy and safety of BKM120 in combination with tamoxifen in patients with ER/PR + , HER2- BC with prior exposure to antihormonal therapy	II	NCT02404844
				To determine the efficacy and safety of treatment with BKM120 plus Fulvestrant *vs.* Placebo plus Fulvestrant in postmenopausal women with HR + , HER2-, AI-treated, locally MBC whose disease progressed on or after mTORi-based treatment	III	NCT01633060
				Consistent, dose-dependent PD activity has been demonstrated and clear signs of anti-tumor activity have been seen with BKM120	I	NCT01513356
			GDC-0941	Examining how well the combination of GDC-0941 and cisplatin work in treating patients with metastatic AR- TNBC	I/II	NCT01918306
				Assessing the safety, tolerability and efficacy of GDC-0032 or GDC-0941, in combination with PAlbociclib, with the subsequent addition of Fulvestrant in *PIK3CA*-mutant BCs	I	NCT02389842
				To assess the safety, tolerability, and PO of pictilisib administered with letrozole or IV paclitaxel with and without IV bevacizumab or IV trastuzumab in participants with locally recurrent or metastatic BC	I	NCT00960960
			GDC 0032	GDC 0032 is given together with enzalutamide and to see how well they work in treating patients with metastasis AR + TNBC	I/II	NCT02457910
				Assessing the safety, tolerability and efficacy of GDC-0032 or GDC-0941, in combination with PAlbociclib, with the subsequent addition of Fulvestrant in *PIK3CA*-mutant BCs	I	NCT02389842
				To determine RP2D of GDC-0032 plus tamoxifen in HR + , HER2-MBC patients who have progressed after prior endocrine treatment	I/II	NCT02285179
			BAY 80–6946	To study the SE and how well BAY 80–6946 works when given together with fulvestrant in treating postmenopausal patients with ER + and HER2- BC that has spread to other places in the body and progressing after prior treatment	I/II	NCT03803761
				The addition of BAY 80–6946 to the usual treatment (trastuzumab and pertuzumab) could shrink the cancer or stabilize it for longer duration as compared to the usual treatment alone	I/II	NCT04108858
				Adding BAY 80–6946 to the usual therapy of Fulvestrant and abemaciclib may work better than giving Fulvestrant and abemaciclib alone in treating patients with BC	I/II	NCT03939897
				BAY 80–6946 may stop the growth of tumor cells by blocking some of the enzymes needed for cell growth	II	NCT03377101
				Giving BAY 80–6946, letrozole, and palbociclib may work better in treating patients with BC	I/II	NCT03128619
		*IS*	BYL719	BYL719 in combination with letrozole may kill more tumor cells	I	NCT01791478
				BYL719 is an oral drug that may help T-DM1 to work better	I	NCT02038010
				Determining the MTD, safety and effectiveness of BYL719 combined with Nab-Paclitaxel in treating patients with HER2-BC, along with the determination of how long this drug combination will keep the disease from getting worse	I/II	NCT02379247
				A Study of BYL719 in combination with paclitaxel in advanced solid tumors followed by two expansion phases in locally chemotherapy naive HER2-MBC patients and in recurrent and metastatic HNSCC patients pre-treated with platinum-based therapy	I	NCT02051751
				To determine whether treatment with BYL719 plus letrozole led to an increase in pathologic clinical response and ORR compared to treatment with placebo plus letrozole in patients with BC	II	NCT01923168
				To assess the MTD and/or the RP2D(s), safety, tolerability, the single and multiple dose PK profile and the preliminary anti-tumor activity of BYL719 and BKM120 in combination with tamoxifen plus goserelin acetate in premenopausal advanced HR + BC patients	I	NCT02058381
				To describe safety and tolerability of the BYL719 and everolimus or BYL719, everolimus and exemestane combinations	I	NCT02077933
				To study BYL719 monotherapy in adult patients with advanced MBC progressing after first line therapy	II	NCT02506556
				BKM120, BYL719 and olaparib are drugs that may stop cancer cells from growing abnormally	I	NCT01623349
				BYL719 may stop the growth of tumor cells by blocking some of the enzymes needed for cell growth	I	NCT03207529
				Assessing the efficacy and safety of BYL719 plus Fulvestrant or letrozole, based on prior endocrine therapy, in patients with *PIK3CA* mutation with advanced BC who have progressed on or after prior treatments	II	NCT03056755
				To investigate combination of BYL719 with Fulvestrant in post-menopausal patients with locally advanced or MBC whose tumors have an alteration of the *PIK3CA* gene	I	NCT01219699
			MEN1611	To identify the appropriate dose of MEN1611 to be used in combination with Trastuzumab with/without Fulvestrant for the treatment of HER2 + MBC	I	NCT03767335
			BAY80-6946	It will determine the MTD and the RP2D of BAY80-6946 in combination with paclitaxel	I	NCT01411410
			XL147	Phase 1 will evaluate the MTD of XL147 or XL765 when given in combination with letrozole. Phase 2 will evaluate the efficacy and safety of these combinations in subjects with BC refractory to a non-steroidal aromatase inhibitor that is ER + /PGR + and HER2-	I/II	NCT01082068
			TAK-117	To test if combining TAK-117 with canagliflozin will improve efficacy in the treatment of advanced solid tumors	I/II	NCT04073680
		*Dual*	BEZ235	Evaluating the safety profile/tolerability and preliminary anti-tumor effect of BKM120 and endocrine therapy combination and BEZ235 and endocrine therapy combination in postmenopausal patients with HR + MBC	I	NCT01248494
				This is a first-in-human, phase I/Ib clinical research study with BEZ235	I	NCT00620594
			CUDC-907	Evaluating the safety, tolerability and PK of CUDC-907 administered orally to subjects with advanced/relapsed solid tumors	I	NCT02307240
			LY3023414	To investigate the safety of prexasertib in combination with other anti-cancer drugs including LY3023414 in participants with advanced or metastatic cancer	I	NCT02124148
			PF-04691502	The combination of PF-04691502 and exemestane might mitigate resistance to hormonal therapy and result in greater clinical benefit than exemestane alone in women with advanced ER + BC	II	NCT01658176
				Published data support the hypothesis that a PF-04691502 in combination with letrozole might mitigate the intrinsic or acquired resistance to hormonal therapy and restore hormone sensitivity in high risk patient population of hormone-sensitive BCs	I	NCT01430585
			PKI-587	Preclinical and first-in-human studies have shown a manageable safety profile with predictable toxicity for this class of drugs	I	NCT02626507
			XL765	Phase 1 will evaluate the MTD of XL147 or XL765 when given in combination with letrozole. Phase 2 will evaluate the efficacy and safety of these combinations in subjects with BC refractory to a non-steroidal aromatase inhibitor that is ER + /PGR + and HER2-	I/II	NCT01082068
			PQR309	To evaluate clinical safety, efficacy and PK of PQR309 in combination with standard dose of eribulin in patients with locally advanced or metastatic HER2-TNBC	I/II	NCT02723877
	OC	*Pan-*	BAY 80–6946	Niraparib and BAY 80–6946 may stop the growth of tumor cells by blocking some of the enzymes needed for cell growth	I	NCT03586661
			BKM120	To look for MTD, and also to see if the combination of BKM120 or BYL719 and olaparib is effective in treating OC	I	NCT01623349
		*IS*	BYL719	To look for MTD, and also to see if the combination of BKM120 or BYL719 and olaparib is effective in treating OC	I	NCT01623349
		*Dual*	CUDC-907	Evaluating the safety, tolerability and PK of CUDC-907 administered orally to subjects with advanced/relapsed solid tumors	I	NCT02307240
	FTC	*Pan-*	BAY 80–6946	Niraparib and BAY 80–6946 may stop the growth of tumor cells by blocking some of the enzymes needed for cell growth	I	NCT03586661
	EC	*Pan-*	BAY 80–6946	Niraparib and BAY 80–6946 may stop the growth of tumor cells by blocking some of the enzymes needed for cell growth	I	NCT03586661
				BAY 80–6946 may stop the growth of tumor cells by blocking some of the enzymes needed for cell growth	II	NCT02728258
		*IS*	TAK-117	To test the hypothesis that combining TAK-117 with canagliflozin will improve efficacy in treating patients with advanced EC	I/II	NCT04073680
			MLN1117	Study of MLN0128, combination of MLN0128 with MLN1117, Paclitaxel and combination of MLN0128 with Paclitaxel in women with EC	II	NCT02725268
		*Dual*	PF-04691502	To investigate the individual safety and efficacy of PF-04691502 in patients with recurrent EC	II	NCT01420081
			PKI-587	To investigate the individual safety and efficacy of PKI-587 in patients with recurrent EC	II	NCT01420081
			LY3023414	To determine the effectiveness and the side effects of LY3023414 in treating the EC	II	NCT02549989
			DS-7423	To determine the MTD in subjects with advanced solid tumors and measure the effects of DS-7423 on the patients with advanced EC	I	NCT01364844
Genitourinary	PCa	*Pan-*	BKM120	To evaluate BKM120 with cabazitaxel in the treatment of patients with advanced PCa	II	NCT02035124
		*IS*	AZD8186	To explore the efficacy of AZD8186 as monotherapy or in combination with abiraterone acetate or AZD2014 in patients with CRPC	I	NCT01884285
			GSK2636771	To determine the RP2D for the combination GSK2636771 with enzalutamide in male subjects with metastatic CRPC	I	NCT02215096
		*Dual*	LY3023414	To evaluate the safety and effectiveness of LY3023414 in combination with enzalutamide in men with PCa	II	NCT02407054
			GDC-0980	Phase Ib is to determine RP2D of ipataseritib administrated in combination with abiraterone and of GDC-0980 administrated in combination with abiraterone	I/II	NCT01485861
	RCC	*IS*	BYL719	To describe safety and tolerability of the BYL719 and everolimus or BYL719, everolimus and exemestane combinations	I	NCT02077933
			MLN1117	To evaluate the efficacy and safety of single-agent MLN0128 and the combination of MLN0128 + MLN1117 compared with everolimus in the treatment of adults with advanced or metastatic Clear-Cell RCC	II	NCT02724020
	BLCA	*Pan-*	BKM120	To learn what effects, good and/or bad, BKM120 has on advanced urothelial cancer	II	NCT01551030
		*IS*	GSK2636771	To evaluate the ORR of patients targeted study agent(s) in patients with advanced refractory cancers	II	NCT02465060
Hematologic	Lymphoma	*Pan-*	BAY80-6946	To study the SE and BD of BAY 80–6946 and nivolumab in treating patients with metastatic solid tumors or lymphoma	I	NCT03502733
				To investigate safe, feasible and beneficial of BAY80-6946 in pediatric patients with recurrent or refractory lymphoma	I/II	NCT03458728
				To evaluate the ORR of patients targeted study agent(s) in patients with advanced refractory cancers	II	NCT02465060
			BKM120	To find out what effects, good and/or bad, BKM120 has on lymphoma and the central nervous system	II	NCT02301364
		*IS*	TGR-1202	Phase I is to determine the MTD, DLT, safety and toxicity of the combinations of TGR-1202 and carfilzomib in participants with R/R NHL and HL. If the combination is found to be feasible, phase II consisting of a 2-stage design of the combination will be initiated	I/II	NCT02867618
			IPI-145	To evaluate the safety and PK of IPI-145 in Japanese participants with R/R lymphoma	I	NCT02598570
				To characterize the safety, MTD and preliminary efficacy profile of IPI-145 given in combination with rituximab, or bendamustine plus rituximab, to subjects with select R/R hematologic malignancies	I	NCT01871675
		*Dual*	PQR309	To determine the MTD, RP2D and preliminary antitumor activity of PQR309 administered orally, as once daily capsules continuously and on intermittent schedule in patients with R/R lymphomas	II	NCT02249429
			VS-5584	To evaluate the safety (including the RP2D), PK and the anti-cancer activity of VS-5584	I	NCT01991938
			WX390	WX390 is a novel oral small molecular that has demonstrated potent inhibitory effects on multiple human tumor xenografts	I	NCT03730142
			GSK1059615	To define the RP2D, toxicity profile, PK and biologically active dose range of GSK1059615	I	NCT00695448
			CUDC-907	To assess the safety, tolerability and PK of orally Administered CUDC-907 in subjects with R/R lymphoma	I/II	NCT01742988
			GSK2126458	To determine the RP2D of GSK2126458 based on safety and tolerability, PK, PD and preliminary evidence of clinical activity	I	NCT00972686
	HL	*IS*	TGR-1202	To evaluate the safety and effectiveness of TGR-1202 in combination with brentuximab vedotin in patients with HL	I	NCT02164006
			RP6530	To evaluate safety, tolerability and to establish the MTD for RP6530 in combination with Pembrolizumab in patients with CHL	I/II	NCT03471351
	NHL	*Pan-*	BAY80-6946	BAY80-6946 in combination with standard immunochemotherapy *vs.* standard immunochemotherapy in patients with relapsed iNHL	III	NCT02626455
				To assess the safety of BAY80-6946 in Rituximab-refractory iNHL	III	NCT02369016
				Part A is to evaluate the efficacy and safety of BAY80-6946 in patients with indolent or aggressive NHL, who have progressed after standard therapy. Part B is to evaluate the efficacy and safety of BAY80-6946 in patients with R/R FL	II	NCT01660451
				To study BD and how well BAY80-6946 plus nivolumab works in patients with Richter's transformation or transformed iNHL	I	NCT03884998
				To study the BD of BAY80-6946 plus chemotherapy in patients with R/R DLBCL or relapsed grade 3b FL after 1 prior line therapy	I	NCT04156828
			BKM120	BKM may stop the growth of cancer cells by blocking some of the enzymes needed for cell growth	I	NCT01719250
			GDC-0941	To assess the safety, tolerability, and PK of orally administered GDC-0941 administered QD	I	NCT00876122
			GDC-0032	To assess the safety, tolerability, and PK of GDC-0032 in participants with NHL	I	NCT01296555
		*IS*	IPI-145	To assess the safety, PK, drug-drug interactions, and RP2D of co administered IPI-145 and Venetoclax in subjects with R/R CLL/SLL or iNHL who have not previously received a Bcl-2 or PI3K Inhibitor	I	NCT02640833
				Examine the effects of predefined 2 weeks IPI-145 dose holidays on tumor responses and safety/tolerability	II	NCT04038359
				To evaluate the safety and efficacy of IPI-145 in subjects with iNHL that is refractory to rituximab and to either chemotherapy or RIT	II	NCT01882803
				To evaluate the efficacy and safety of DBR vs PBR in subjects with previously-treated iNHL	III	NCT02576275
			CAL-101	To evaluate the efficacy, safety, tolerability, and PD of entospletinib and CAL-101	II	NCT01796470
				To evaluate the addition of CAL-101 to bendamustine/rituximab on PFS in adults with previously treated iNHL	III	NCT01732926
			RP6530	To assess the anti-tumor activity and safety of RP6530 in patients with R/R iNHL	II	NCT03711578
			TGR-1202	To evaluate the safety and effectiveness of Ublituximab in combination with TGR-1202, with or without ibrutinib or bendamustine, in patients with advanced hematologic malignancies	I	NCT02006485
		*Dual*	PQR309	With a safety run-in evaluating efficacy and safety of PQR309 in patients with R/R Lymphoma	II	NCT03127020
		*Dual*	LY3023414	To study how well LY3023414 works in treating patients with recurrent NHL	II	NCT03213678
	BCL	*Pan-*	BKM120	BKM120 may stop the growth of cancer cells and when it together with rituximab may be an effective treatment for BCL	I	NCT02049541
			BAY80-6946	To evaluate whether copanlisib plus rituximab is superior to placebo plus rituximab in prolonging PFS in patients with relapsed iNHL	III	NCT02367040
				To establish the MTD and RP2D of BAY80-6946 in combination with venetoclax in patients with R/R B-cell NHL	I	NCT03886649
		*IS*	CAL-101	To evaluate the safety of CAL-101 as post-transplantation maintenance in patients with BCL undergoing an allogeneic HSCT	I	NCT03151057
				To assess the overall response rate, the efficacy and safety of CAL-101 in participants with previously treated iNHL that is refractory both to rituximab and to alkylating-agent-containing chemotherapy	II	NCT01282424
			RP6530	To evaluate the safety and efficacy of RP6530 in patients with hematologic malignancies	I	NCT02017613
			KA2237	To evaluate safety/tolerability, PK and PD effects of KA2237 in patients with BCL and determine the MTD in Part I of the study. In Part II, patients with BCL will be treated with KA2237 at the MTD to evaluate safety and efficacy in the patient population	I	NCT02679196
			YY-20394	To assess the tolerability, PK and efficacy of YY-20394 in patients with relapse or refractory BCL	I	NCT03757000
			TGR-1202	TGR-1202 and ibrutinib may stop the growth of cancer cells by blocking some of the enzymes needed for cell growth	II	NCT02874404
	TCL	*IS*	RP6530	Evaluating the safety, PK and efficacy of RP6530 in patients with R/R TCL	I	NCT02567656
				To evaluate the safety and efficacy of RP6530 in patients with hematologic malignancies	I	NCT02017613
				To characterize safety, tolerability and to establish the MTD of RP6530 in combination with Romidepsin in patients with R/R TCL	I/II	NCT03770000
			IPI-145	This is a study of IPI-145 in patients with R/R PTCL	II	NCT03372057
				To determine the MTD of IPI-145 with romidepsin and IPI-145 with bortezomib in R/R TCL	I	NCT02783625
	FL	*Pan-*	BAY80-6946	Part B is to evaluate the efficacy and safety of BAY80-6946 in patients with R/R FL	II	NCT01660451
				To see if BAY80-6946 plus rituximab is effective at slowing the growth of FL	II	NCT03789240
		*IS*	IPI-145	To evaluate the safety and efficacy of IPI-145 administered in combination with rituximab vs. placebo in combination with rituximab in patients with previously treated CD20 + FL who are not suitable candidates for chemotherapy	III	NCT02204982
				To evaluate the safety and efficacy of IPI-145 in combination with rituximab or obinutuzumab in subjects with untreated CD20 + FL	I/II	NCT02391545
			TGR-1202	To determine the overall response rate of TGR-1202 in FL	II	NCT03178201
			CAL-101	To establish a safe and effective dosing regimen of CAL-101 in participants with R/R FL who have no other therapeutic options	III	NCT02536300
			INCB050465	To assess the ORR of INCB050465 treatment in subjects with R/R FL	II	NCT03126019
			ME-401	A Three-Arm Study of ME-401 in subjects with R/R FL or CLL/SLL	I	NCT02914938
	NK/TCL	*Pan-*	BAY80-6946	BAY 80–6946 has demonstrated activity in R/R, aggressive NHLs, suggesting an ORR of 50% for TCL. BAY 80–6946 plus gemcitabine will exhibit early elimination of rapidly growing tumor cells and be a rational therapeutic modality for use in R/R PTCLs, if the overlapping toxicities can be managed	I/II	NCT03052933
	CLL/SLL	*Pan-*	BAY80-6946	To study how well bendamustine and rituximab in combination with BAY80-6946 work in treating patients with CLL/SLL	II	NCT04155840
			BKM120	To find out the effects of BKM120 in CLL	II	NCT02340780
		*IS*	CAL-101	The CLL2-BCG-trial is a prospective, open-label, multicenter phase-II-trial	II	NCT02445131
				To evaluate a combination of drugs called Ofatumumab and CAL-101 as a possible treatment for CLL and SLL	II	NCT02135133
				To determine the preliminary efficacy and safety of the combination of tirabrutinib and CAL-101 with obinutuzumab in adults with R/R CLL	II	NCT02968563
			IPI-145	To study IPI-145 and Venetoclax in subjects with R/R CLL/SLL or iNHL who have not previously received a Bcl-2 or PI3K Inhibitor	I	NCT02640833
				To examine the efficacy of IPI-145 monotherapy *vs.* ofatumumab monotherapy in subjects with R/R CLL/SLL	III	NCT02004522
				To examine the efficacy of IPI-145 monotherapy or ofatumumab monotherapy in subjects with CLL/SLL who experienced disease progression after treatment with IPI-145 or ofatumumab in study IPI-145–07	III	NCT02049515
				To study IPI-145 in patients with CLL/SLL who have previously been treated with ibrutinib or another BTK Inhibitor and R/R to such therapy or discontinued such therapy due to toxicity	II	NCT03370185
				To test safety, PK and PD of IPI-145 in combination with obinutuzumab in patients with CLL/SLL previously treated with a BTKi	I	NCT02292225
			TGR-1202	TGR-1202 may stop cancer cells from growing and this drug may help to kill cancer cells when coupled with ibrutinib	I	NCT02268851
				A study of TGR-1202 administered as a single agent in CLL patients who are intolerant to prior BTKi or prior PI3Kδ inhibitors	II	NCT02742090
	MCL	*IS*	INCB050465	Evaluating efficacy and safety of 2 INCB050465 treatment regimens in subjects with R/R MCL treated either with or without a BTKi	II	NCT03235544
	MZL	*Pan-*	BAY80-6946	To test the toxicity and efficacy of BAY 80–6946 in combination with Rituximab in patients with newly diagnosed or relapsed MZL	II	NCT03474744
		*IS*	INCB050465	To study INCB050465 in subjects with R/R MZL with or without prior exposure to CITADEL-204	II	NCT03144674
	DLBCL	*Pan-*	BAY80-6946	To study how well BAY 80–6946 hydrochloride and nivolumab work in treating patients with R/R DLBCL or PMLBC	II	NCT03484819
				To assess efficacy of BAY80-6946 in R/R DLBCL patients and the relationship between efficacy and a predictive biomarker	II	NCT02391116
		*IS*	INCB050465	To assess the safety and efficacy of INCB050465 in subjects with R/R DLBCL	II	NCT02998476
				To evaluate the safety and tolerability of INCB053914 in combination with INCB050465 in R/R DLBCL	I	NCT03688152
		*Dual*	CUDC-907	To evaluate the efficacy and safety of CUDC-907 in subjects 18 years and older with R/R MYC-altered DLBCL	II	NCT02674750
	PCNSL	*Pan-*	BAY80-6946	To test the safety of combined use of the study drugs, BAY80-6946 and ibrutinib, in people with PCNSL	I/ II	NCT03581942
	MM	*IS*	BYL719	To estimate the MTD and/or RP2D of the combination of LGH447 and BYL719 administered orally to adult patients with R/R MM	I	NCT02144038
	Leukemia	*Pan-*	BKM120	To find the MTD of BKM120 that can be given to patients with R/R leukemia	I	NCT01396499
		*IS*	CAL-101	To provide CAL-101 to individuals with relapsed, previously treated CLL who have limited treatment options		NCT02136511
				To evaluate the effect of the addition of CAL-101 to bendamustine + rituximab on PFS in participants with previously treated CLL	III	NCT01569295
				To evaluate the effect of idelalisib in combination with rituximab on the onset, magnitude, and duration of tumor control in participants previously treated for CLL	III	NCT01539512
				To evaluate the effectiveness of CAL-101 and rituximab in adults with CLL in a real world setting		NCT03582098
				Obtaining more in-depth information on how patients with CLL treated with CAL-101 and rituximab react to treatment		NCT03545035
				To study how well pembrolizumab alone or with CAL-101 or ibrutinib works in treating patients with CLL or other iB-NHL	II	NCT02332980
				To evaluate efficacy, safety, tolerability and PD of entospletinib and CAL-101 in patients with CLL, FL, MCL, DLBCL, or iB-NHL	II	NCT01796470
				To confirm the hypothesis that CAL-101 may represent a new therapeutic alternative for patients with ALL in a set of particularly complex scenarios: relapsed, refractory to conventional treatments, and old age	I/II	NCT03742323
				To investigate the safety and clinical activity of CAL-101 in combination with chemotherapeutic agents, immunomodulatory agents and anti-CD20 mAb in subjects with R/R iNHL, MCL or CLL	I	NCT01088048
				To investigate the safety, PK, PD, and clinical activity of CAL-101 in patients with select, R/R Hematologic Malignancies	I	NCT00710528
				To determine how well the test can be used to select personalized kinase inhibitor therapy in combination with standard chemotherapy in treating patients with newly diagnosed AML and ALL	I	NCT02779283
			YY-20394	To assess the tolerability, PK and efficacy of YY-20394 in patients with relapse or refractory B cell malignant hematological tumor	I	NCT03757000
		*Dual*	BEZ235	To establish the MTD and the RP2D of BEZ235 when administered twice daily as a single agent in patients with R/R acute leukemia	I	NCT01756118
			PKI-587	Phase II open-label single-arm prospective multicentric clinical trial of PKI-587 delivered by intravenous route	II	NCT02438761
Bone and soft tissue	OS or EWS	*Pan-*	BAY80-6946	To investigate safe, feasible and beneficial of BAY80-6946 in pediatric patients with recurrent or refractory OS, EWS or lymphoma	I/II	NCT03458728
		*Dual*	LY3023414	To study how well LY3023414 works in treating patients with recurrent OS, EWS or NHL	II	NCT03213678
Skin	Melanoma	*Pan-*	BKM120	Trial of BKM120 in patients with metastatic melanoma with brain metastases who are not eligible for surgery or radiosurgery	II	NCT02452294
			PX-866	Phase 1/2 study of PX-866 combined with Vemurafenib in patients with *BRAF*-mutant cancer including advanced melanoma	I/II	NCT01616199
		*IS*	GSK2636771	To learn if GSK2636771 given in combination with pembrolizumab can help to control the disease in patients with refractory metastatic melanoma	I/II	NCT03131908

*AI* aromatase inhibitor, *ALL* acute lymphoblastic leukemia, *AML* acute myeloid leukemia, *BC* breast cancer, *BCL* B-cell lymphoma, *BD* best dose, *BLCA* bladder cancer, *BTKi* BTK inhibitors, *CHL* classical Hodgkin lymphoma, *CLL/SLL* chronic lymphocytic leukemia or small lymphocytic lymphoma, *CRC* colorectal carcinoma, *CRPC* Castration-Resistant Prostate Cancer, *DBR* duvelisib in combination with bendamustine and rituximab, *DLT* dose limiting toxicity, *EC* endometrial cancer, *ESCC* esophageal squamous cell carcinoma, *EWS* Ewing's sarcoma, *FL* follicular lymphoma, *FTC* fallopian tube carcinoma, *GBM* Glioblastoma multiforme, *GC* gastric cancer, *GIST* gastrointestinal stromal tumor, *HCC* hepatocellular carcinoma, *HL* Hodgkin's lymphoma, *HNSCC* head-and-neck squamous cell carcinoma, *HR* hormone receptor, *HSCT* hematopoietic stem cell transplant, *iB-NHL* indolent B cell non-Hodgkin's lymphoma, *iNHL* indolent non-Hodgkin's lymphoma, *IS* isoform-selective, *IV* intravenous, *KC* kidney cancer, *MBC* metastatic Breast Cancer, *MBM* medulloblastoma, *MCL* mantle cell lymphoma, *MM* multiple myeloma, *MRD* maximum recommended dose, *MTD* maximum tolerated dose, *mTORi* rapamycin inhibitor, *MZL* marginal zone lymphoma, *NHL* non-Hodgkin's lymphoma, *NSCLC* non-small cell lung cancer, *NK/TCL* NK/T cell lymphomas, *OC* ovarian cancer, *ORR* objective response rate, *OS* osteosarcoma, *PBR* placebo in combination with bendamustine and rituximab, *PC* pancreatic cancer, *PCa* prostate cancer, *PCNSL* primary central nervous system lymphoma, *PD* pharmacodynamics, *PDAC* pancreatic ductal adenocarcinoma, *PFS* progression-free survival, *PK* pharmacokinetics, *PMLBCL* primary mediastinal large B-cell lymphoma, *PNETs* pancreatic neuroendocine neoplasms, *PO* pharmacokinetics of oral, *PTCL* peripheral T-cell lymphoma, *RCC* renal cell cancer, *RIT* radioimmunotherapy, *RP2D* recommended phase 2 dose, *R/R* relapsed and/ or refractory, *SCCHN* squamous cell carcinoma of the head and neck, *SCLC* small cell lung cancer, *SE* side effects, *TC* thyroid cancers, *TCL* T-cell lymphoma, *TNBC* triple negative breast cancer, *UM* uveal melanoma

By the way, although AKT isoforms are observed to play different roles in GBM, including AKT3 delays tumor progression [27], as a matter of fact, the AKT inhibitor perifosine is tolerable but ineffective as monotherapy for GBM [28]. AKT inhibitors remain elusive and bear the weight of further examination in treating GBM.

Notably, building on that 22% genetic alterations of *PTEN* was detected in GBM (https://www.cbioportal.org, Table 1), especially deep deletion, which caused the loss of function of PTEN tumor suppressor, PTEN was deeply involved in the pathological effects of PI3K/AKT pathway in GBM [29]. Meanwhile, genetic loss of *PTEN* is associated with each subtype of GBM [30].

Additionally, glucose regulated protein 78 (GRP78) interacts with α2-macroglobulin to activate AKT1 via PDK1, as well as mTOR to enhance cancer cell proliferation and radiotherapy resistance in GBM [31–33]. Anti-GRP 78 antibody can restore cancer cells to sensitivity to radiation therapy, which inhibits cell proliferation and enhances apoptosis, and has the advantage of targeting against cancer cells without affecting normal cells. Moreover, combination of anti-GRP 78 antibody and radiation therapy (XRT) shows better inhibitory effect on tumor [31].

Compared to GBM, the genetic alteration of *PIK3CA* (2%) and *PIK3R1* (0.3%) in medulloblastoma (MBM, Table 1), which is the most aggressive malignant brain tumor that highly occurs in children and survival rate can reach 70% after active treatment, are less frequently observed [34]. However, enhance phosphorylation of AKT via PI3K or mTOR to restrain GSK3 in MBM, which lead to SOX9 degradation is reduced due to the facts that FBW7 degrades SOX9 under the guidance of GSK3. The loss of FBW7 function increases SOX9 protein levels, increasing the malignancy of cancer and resistance to cisplatin [35]. As a major oncoprotein inhibitor, once FBW7 is deleted or mutated, it can cause tumors to occur directly [36, 37]. So targeted inhibition of the PI3K pathway has a bright therapeutic potential in MBM. Moreover, experiments show that combination of PI3K inhibitor, mTOR inhibitor and cisplatin can achieve better therapeutic effect [35], and how well LY3023414 works in recurrent MBM is being tested in an ongoing clinical trial (NCT03213678, Table 2).

### Aberration of the PI3K/AKT pathway in the cancer of endocrine system

Thyroid cancer (TC) is the most common malignancy in the endocrine system with a global incidence rate of 3.1% but a relatively lower lethality (0.4%, Table 1). In view of the fact that follicular epithelial cell–derived TC accounts for > 95% of all thyroid malignancies, TC histologically comprises papillary thyroid cancer (PTC), follicular thyroid cancer (FTC), poorly differentiated thyroid cancer (PDTC) and anaplastic thyroid cancer (ATC) [38]. Although PDTC and ATC only account for approximately 5%–10% of TC, but they have brought great clinical challenges since they beget two-thirds of TC-related deaths [39]. Obviously, the overall genetic alterations of PI3K/AKT pathway in TC are inconspicuous (Table 1), but genetic mutations in PI3K/AKT pathway are common in PDTC and ATC, specifically more common in ATC than in PDTC. Besides *PIK3CA* (18% vs. 2%) and *PTEN* (15% vs. 4%), mutations of *PIK3C2G* (6% vs. 1%), *PIK3CG* (6% vs. 1%), *PIK3C3* (0 vs. 1%), *PIK3R1* (0 vs. 1%), *PIK3R2* (3% vs. 0), *AKT3* (0 vs. 1%) are also observed in ATC and PDTC respectively [40]. REC8, TEKT4, ING5, c-Met, HPIP, PIG3, TBX1, CRLF1, INPP4B, MAPK4, miR-34a, -125b, -126, -145, -146b, -148a and -766, as well as lncRNA LINC003121, ABHD11-AS1, H19 and XIST regulate TC cell growth, tumor progression, migration, metastasis or epithelial–mesenchymal transition (EMT) through activating PI3K/AKT pathway [41–61]. Actually, exclusive activating mutations of *BRAF* (60% vs. 33% and 38%) in PTC are more frequently observed than in PDTC and ATC [40], while mice experiments show that co-mutation of *BRAF* and *PIK3CA* can promote the development of lethal ATC, but neither *BRAF* nor *PIK3CA* mutations alone can [62]. In addition, mutations in *BRAF* and *PIK3CA* can activate the MAPK pathway and the PI3K/AKT pathway respectively and lead to the occurrence of ATC, whereas dual blocking PI3K and MAPK pathways can effectively inhibit ATC [63]. Dual PI3K/HDAC inhibitor CUDC-907 inhibits TC growth and metastases, and may be a promising treatment strategy for advanced, metastatic TC [64]. Moreover, whether CUDC-907 was safe and effective in ATC and PDTC patients had been attempted in a terminated clinical trial (NCT03002623) besides the clinical trial of BKM120 in patients with advanced or metastatic differentiated TCs (NCT01830504, Table 2).

### Characterization of the PI3K/AKT pathway in the respiratory system tumor

The respiratory system tumors are composed of the upper respiratory tract tumors, such as nasopharyngeal carcinoma (NPC) and laryngeal cancer, and the lower respiratory tract tumors, which mainly refer to LC. Compared to the NPC and laryngeal cancer, LC is witnessed as the gender-free and world-wide cancer with the highest morbidity (11.6%) and mortality (18.4%, Table 1).

LC is classified into two categories: small cell lung cancer (SCLC) and non-small cell lung cancer (NSCLC) including three subtypes: adenocarcinoma (ADC), squamous cell carcinoma (SCC) and large cell carcinoma (LCC) [65]. In the light of the fact that genetic alterations of *PIK3CA* (3% vs. 17%), *PIK3R1* (2% vs. 1.8%), *PIK3R2* (1.5% vs. 1.6%), *AKT1* (0.5% vs. 2.1%), *AKT2* (1.5% vs. 3%) and *PTEN* (8% vs. 6%) are observed in SCLC and NSCLC respectively (Table 1), the studies of treatment strategies of LC targeting PI3K/AKT pathway are in full swing. Apart from those widely recognized alterations, such as *EGFR* and *KRAS* gene mutations, *MET* amplification, *EML4-ALK* rearrangements in NSCLC, somatic mutations and amplification in *PIK3CA* are described in 3–10% vs. 35% of SCC and 0–2.7% vs. 7% of ADC respectively [66]. What’s more, the expression of PIK3IP1, a negative regulator of PI3K, which can combine the p110 catalytic subunit of PI3K heterodimers to inhibits the activity of PI3K catalytic, is significantly lower in ADC and other tumors tissues [67]. ROCK1, GPX1, PAX6-ZEB2 axis, miR-93 and -496, as well as LINC00665 participate in regulation of the growth, migration, tumorigenesis or chemoresistance of NSCLC through PI3K/AKT pathway [68–73]. Furthermore, IGF-1 activates PI3K/AKT/β-catenin axis, which promotes the symmetric cell division of lung CSC and expands CSC pool, to maintain tumorigenesis [74, 75]. Interestingly, GRP78 plays the same role in radiation resistance and survival of cells in NSCLC by activating AKT1 as in GBM [31]. Currently, the potential of PI3K/AKT inhibitors has been clinically evaluated in a considerable number of studies (Tables 2 and 3) with NSCLC patients. On the other hand, MCAM and EPHA3 mediate chemoresistance in SCLC via the PI3K/AKT pathway [76, 77]. Whether combining daily BKM120 with cisplatin and etoposide was safe and effective in extensive stage SCLC patients had been attempted in a completed clinical trial (NCT02194049, Table 3).

**Table 3:** Clinical trial of AKT Inhibitors in cancers (as of December 2019) (https://clinicaltrials.gov)

System	Cancer	Subunit	Inhibitors	Characteristic	Clinical trials	
					Phase	Gov identifier
Brain and central nervous	GBM	Allosteric	Perifosine	30 adults with recurrent GBM were treated with a loading dose of 600 mg Perifosine followed by 100 mg daily until either disease progression or intolerable toxicity. Perifosine is tolerable but ineffective as monotherapy for GBM. Preclinical data suggests synergistic effects of Perifosine in combination with other approaches, and further study is ongoing	II	(24)
	UM	ATP-comp	GSK2141795	Trametinib and GSK2141795 may stop the growth of tumor cells by blocking some of the enzymes needed for cell growth. It is not yet known whether trametinib is more effective with or without GSK2141795 in treating patients with metastatic UM	II	NCT01979523
Respiratory	NSCLC	Allosteric	MK2206	MK2206 and erlotinib hydrochloride may stop the growth of tumor cells by blocking some of the enzymes needed for cell growth	II	NCT01294306
				Combination of MK2206 and gefitinib for the treatment of patients with NCLC who have failed prior chemotherapy and an EGFR-TKI	I	NCT01147211
				Whether it helps to control NSCLC with drug combinations (Erlotinib + MK2206 or AZD6244 + MK2206) and the safety of these drug combinations remains to be studied	II	NCT01248247
			Perifosine	To determine the MTD of perifosine that can be administered to people without gastrointestinal toxicity and obtain preliminary information on the response rate of perifosine in NSCLC	I/II	NCT00399789
Digestive	GC	Allosteric	MK2206	To study how well MK2206 works in treating patients with advanced GC or GEJC	II	NCT01260701
				To study the side effects and BD of MK2206 and lapatinib ditosylate when given together with trastuzumab in treating patients with locally advanced or metastatic GC, or GEC that cannot be removed by surgery	I	NCT01705340
		ATP-comp	GSK2110183	To determine the MTD and RP2D for the combination of GSK2110183 and paclitaxel in subjects with recurrent HER2-GC, and further assess safety and preliminary efficacy of combination at the RP2D	I	NCT02240212
			GDC-0068	To evaluate the efficacy of GDC-0068 in combination with oxaliplatin, 5-fluorouracil, and leucovorin chemotherapy in participants with advanced or metastatic GC or GEJC	II	NCT01896531
	CRC	Allosteric	MK2206	To evaluate the safety and effectiveness of MK-2206 and AZD6244 in individuals with advanced CRC that has not responded to standard treatments	II	NCT01333475
				To study how well MK2206 works in treating patients with previously treated CRC that has spread from the primary site to other places in the body or nearby tissue or lymph nodes and cannot be removed by surgery	II	NCT01802320
				MK2206 is being tested in a subgroup of patients with CRC whose tumors have changes in certain genes that may make them more likely to respond to MK2206	II	NCT01186705
		ATP-comp	GSK2141795	GSK2141795 given together with dabrafenib and trametinib may be a better treatment for cancer	I/II	NCT01902173
	HCC	Allosteric	MK2206	MK2206 may stop the growth of tumor cells by blocking some of the enzymes needed for cell growth	II	NCT01239355
				How well MK2206 works in treating patients with advanced or non-resectable HCC	II	NCT01425879
	GBC	Allosteric	MK2206	To study how well selumetinib and MK2206 work in treating patients with refractory or advanced GBC that cannot be removed by surgery	II	NCT01859182
				How well MK2206 works in treating patients with Stage IV GBC	II	NCT01425879
	PC	Allosteric	MK2206	Selumetinib and MK2206 may stop the growth of tumor cells. To find if selumetinib and MK2206 are more effective than oxaliplatin and fluorouracil in treating patients with metastatic PC	II	NCT01658943
Female Reproductive	BC	Allosteric	MK2206	To study how well MK2206 works in treating patients with BC that has spread to other places in the body and usually cannot be cured or controlled with treatment	II	NCT01277757
				To study the side effects and BD of MK2206 when given together with paclitaxel and to see how well they work in treating patients with MBC	I	NCT01263145
				Giving MK2206 together with anastrozole, fulvestrant may kill more tumor cells	I	NCT01344031
				Giving MK-2206, anastrozole, and goserelin acetate together may kill more tumor cells	II	NCT01776008
				MK2206 may stop the growth of MBC cells by blocking some of the enzymes needed for cell growth when combined with Lapatinib ditosylate	I	NCT01281163
				To study the side effects and BD of MK2206 and lapatinib ditosylate when given together with trastuzumab in treating patients with locally advanced or metastatic HER2 + BC that cannot be removed by surgery	I	NCT01705340
		ATP-comp	GSK2141795	Trametinib and GSK2141795 may stop the growth of tumor cells by blocking some of the enzymes needed for cell growth	II	NCT01964924
			AZD5363	AZD5363 may stop the growth of tumor cells by blocking some of the enzymes needed for advanced BC cell growth	I	NCT02077569
				AZD5363 in combination with paclitaxel can be used in triple negative advanced or MBC	II	NCT02423603
			GDC-0068	Combine GDC-0068 with paclitaxel chemotherapy to treat BC	II	NCT02301988
		Indirect^a^	ONC201	ONC201 is able to target tumor cells to get rid of them without affecting normal cells. Giving ONC201 and a MR diet may work better in treating participants with BC	I	NCT03733119
	OC	Allosteric	Perifosine	Perifosine may help docetaxel be more effective in causing cancer cells to die	I	NCT00431054
			Triciribine	Investigate the safety and tolerability, and determine the maximum tolerated dose of triciribine when combined with carboplatin in women with platinum-resistant, recurrent or persistent OC	I/II	NCT01690468
			MK2206	How effective MK2206 is in treating OC with mutations in PI3K/AKT or low levels of PTEN	II	NCT01283035
		ATP-comp	AZD5363	Olaparib and AZD5363 may stop the growth of tumor cells by blocking some of the enzymes needed for cell growth	I/II	NCT02208375
			GSK2110183	GSK2110183 in combination with carboplatin and paclitaxel for the treatment of recurrent platinum-resistant OC	I/II	NCT01653912
			GSK2141795	GSK2141795 given together with dabrafenib and trametinib may be a better treatment for cancer	I/II	NCT01902173
				Investigate the PK and PD of GSK2141795 by18F FDG PET Analysis	I	NCT01266954
	FTC	Allosteric	MK2206	How effective MK-2206 is in treating FTC where there are mutations in *PI3K* or *AKT* or low levels of PTEN	II	NCT01283035
		ATP-comp	AZD5363	Olaparib and AZD5363 may stop the growth of tumor cells by blocking some of the enzymes needed for cell growth	I/II	NCT02208375
	EC	ATP-comp	ARQ 092	Whether ARQ 092 and anastrozole can treat EC remains to be studied	I/II	NCT02476955
			GSK2141795	Trametinib and GSK2141795 may stop the growth of tumor cells. It is not yet known whether trametinib is a more effective treatment for EC when given with or without GSK2141795	I	NCT01935973
		Allosteric	MK2206	MK2206 may stop the growth of EC cells by blocking some of the enzymes needed for cell growth	II	NCT01307631
	CC	ATP-comp	GSK2141795	To evaluate the combination of GSK1120212 and GSK2141795 as a possible treatment for recurrent or persistent CC	II	NCT01958112
Genitourinary	KC	Allosteric	MK2206	To study the side effects and the BD of MK2206 together with hydroxychloroquine in treating patients with advanced KC	I	NCT01480154
				To study the side effects and how well MK2206 or everolimus works in treating patients with KC that does not respond to treatment	II	NCT01239342
	PCa	Allosteric	MK2206	To study the side effects and the BD of MK2206 together with hydroxychloroquine in treating patients with advanced PCa	I	NCT01480154
				To study the side effects and BD of dinaciclib and MK2206 in treating patients with PCa that cannot be removed by surgery	I	NCT01783171
Hematologic	HM	ATP-comp	GSK2110183	To investigate the safety, tolerability, PK, and PD of GSK2110183 in subjects with any HM	I/II	NCT00881946
	Lymphoma	Allosteric	MK2206	To study how well MK2206 works in treating patients with relapsed lymphoma	II	NCT01258998
		ATP-comp	GSK690693	To investigate the safety, tolerability, PK, and PD of GSK690693 given on various schedules in subjects with solid tumors or lymphoma	I	NCT00493818
	NHL	Indirect^a^	ONC201	ONC201 may stop the growth of cancer cells by blocking some of the enzymes needed for cell growth	I/II	NCT02420795
	DLBCL	Allosteric	MK2206	MK2206 may stop the growth of cancer cells by blocking some of the enzymes needed for cell growth	II	NCT01481129
	MM	ATP-comp	GSK2141795	Studying how well trametinib and GSK2141795 work in treating patients with relapsed/refractory MM	II	*NCT01989598*
			GSK2110183	To evaluate safety, tolerability, PK, PD and clinical activity of GSK2110183 dosed in combination with bortezomib and dexamethasone in MM subjects who have failed at least one line of systemic treatment	I	NCT01428492
				To investigate the safety, PK, PD, and clinical activity of GSK1120212 in combination with GSK2110183 in MM patients	I	NCT01476137
		Allosteric	KRX-0401	To assess the efficacy and safety of KRX-0401, Bortezomib and Dexamethasone in MM patients	III	NCT01002248
	Leukemia	Allosteric	MK2206	To study the SE, best way to give, and BD of MK2206 in treating patients with recurrent or refractory solid tumors or leukemia	I	NCT01231919
	AML	ATP-comp	GSK2141795	To study how well trametinib and GSK2141795 work in treating patients with AML	II	NCT01907815
		Allosteric	MK2206	Studying how well MK2206 works in treating patients with relapsed or refractory AML	II	NCT01253447
	CLL/SLL	Allosteric	MK2206	Giving MK2206 with bendamustine hydrochloride and rituximab may be an effective treatment for relapsed CLL/SLL	I/II	NCT01369849
Skin	Melanoma	ATP-comp	GSK2141795	GSK2141795 given together with dabrafenib and trametinib may be a better treatment for cancer	I/II	NCT01902173
		Allosteric	MK2206	To study the side effects and BD of MK2206 together with hydroxychloroquine in treating patients with advanced melanoma	I	NCT01480154
				To study how well selumetinib and MK2206 works in treating patients with stage III or stage IV melanoma who failed prior therapy with vemurafenib or dabrafenib	II	NCT01519427
Solid tumors with *PIK3CA/AKT/PTEN* mutations		Allosteric	ARQ 751	ARQ 751 inhibits the abnormalities of AKT caused by other genes, which prevent or slow the spread of cancer, in addition to ARQ 751 in combination with paclitaxel or fulvestrant may enhance the effect of monotherapy on *PIK3CA/AKT/PTEN* mutation-driven tumors	I	NCT02761694

*AML* acute myeloid leukemia, *Allosteric* Allosteric inhibitor, *ATP-comp* ATP-competitive inhibitor, *BC* breast cancer, *BD* best dose, *BLCA* bladder cancer, *CC* cervical cancer, *CLL/SLL* chronic lymphocytic leukemia or small lymphocytic lymphoma,. *CRC* colorectal carcinoma, *DLBCL *diffuse large B cell lymphoma, *EC* endometrial cancer, *EGFR-TKI* epidermal growth factor receptor tyrosine kinase inhibitor, *FTC* fallopian tube carcinoma, *GBC* gallbladder cancer, *GC* gastric cancer, *GEC* gastroesophageal cancers, *GEJC* gastroesophageal junction cancer, *HCC* hepatocellular carcinoma, *HM* hematologic malignancies, *KC* kidney cancer, *MBC* metastatic breast cancer, *MR* methionine-restricted, *MTD* maximum tolerated dose, *MM* multiple myeloma, *NHL* non-Hodgkin's lymphoma, *NSCLC* non-small cell lung cancer, *OC* ovarian cancer, *PC* pancreatic cancer, *PCa* prostate cancer, *PD* pharmacodynamics, *PFS* progression-free survival, *PK* pharmacokinetics, *RP2D* recommended phase 2 dose; SE, side effects; UM, uveal melanoma

^a^Indirect: ONC201 is an AKT/ERK inhibitor

NPC is a unique cancer prevalent in South-East Asia with strong etiological association with Epstein–Barr virus (EBV) exposure [78]. As expected, NPC has a relatively lower mutational burdens with *PIK3CA* mutations of 1.8% (Table 1), however, there are still numerous of researches involved in PI3K/AKT pathway in NPC. Not only is hyperactivation of PI3K/AKT pathway in relation to NPC progression and prognosis [79], but FOXO1, CHL1, PNUTS, VPS33B interacts with NESG1, RBM3, ARHGAP42 and LncRNA ZFAS1 also display their influence on the proliferation, growth, invasion, metastasis, EMT, chemosensitivity or radio-resistance of NPC cells via PI3K/AKT pathway [80–86]. Moreover, miR-205-5p induces EMT by targeting PTEN via PI3K/AKT pathway in cisplatin-resistant NPC cells [87].

Typically presenting as a form of squamous cell carcinoma, laryngeal cancer is one of common malignancies in the head and neck, which is partly associated with human papillomavirus (HPV) [78, 88, 89]. A series of studies show the mutational events of PI3K pathway (30.5%) in 151 head and neck squamous cell carcinomas (HNSCCs) containing 29 laryngeal squamous cell carcinomas (LSCCs), particularly *PIK3CA* mutations of 12.6% [90–92]. Furthermore, profiling 279 HNSCCs containing 72 LSCCs, alteration events of *PIK3CA* (34% vs. 56%), *PIK3R1* (1 vs. 3%) and *PTEN* (12% vs. 6%) are displayed in 243 HPV (−) and 36 HPV (+) HNSCCs respectively [93]. Additionally, MMP2/3, MEOX2, miR-145 and -138 regulate the growth, apoptosis or migration of LSCC cells by targeting the PI3K/AKT pathway [94–97].

Herein, clinical trials of BKM120 (Table 2) in NPC and LSCC patients may provide the feasibility of new treatment strategies. Even more, the safety and efficacy of AKT inhibitor MK2206 in NPC patients had been evaluated in a completed clinical trial (Table 3).

### Deregulation of the PI3K/AKT pathway in digestive system tumors

It’s well established that the global health status is jeopardized by digestive system tumors, and the incidence and mortality rate of main digestive system tumors including esophageal cancer (ESCA), GC, colorectal cancer (CRC), as well as hepatocellular, gallbladder and pancreatic cancer (PC) are listed in Table 1.

Esophageal squamous cell carcinoma (ESCC) is the most frequent ESCA subtype internationally. In general, the genetic alterations of *PIK3CA* (24%), and *PTEN* (7%) are observed in ESCA (Table 1), especially the somatic mutations of *PIK3CA* (7.2% vs 12.5%), *PIK3C2A* (0.7% vs. 0), *PIK3CG* (2.9% vs. 4.2%) and *PIK3C2G* (0 vs. 37.5%) are observed respectively in 139 paired ESCC cases and 24 cell lines [98]. Even more, *PIK3CA* mutations are frequent in ESCC associated with chagasic megaesophagus and are associated with a worse patient outcome [99]. HERG1, LSD1, CEP55, CACNA2D3, CircVRK1 and lncRNA GAS5 affect the proliferation, migration, invasion or radioresistance of ESCC cells via the PI3K/AKT pathway [100–105]. After all, a limited number of clinical trials of PI3K inhibitors BYL719 and BKM120 in ESCC patients may bring efficacious therapeutic proposals (Table 2).

The incidence (5.7%) of GC, in which gastric adenocarcinoma (GAC) is the dominant subtype, has continued to decline worldwide due to the *H pylori* treatment [106], but the mortality rate (8.2%) remains the second most common cause of cancer death worldwide (Table 1). As shown in Table 1, the overall genetic alterations of PI3K/AKT pathway are observed with *PIK3CA* (17%) and *PTEN* (11%) in GC. But one research reveals that PI3K/AKT pathway genetic mutations are found in 69 (16%) of the 431 GC patients including *PIK3CA* (13.2%) and *PTEN* (4.0%), as well as *PIK3CA* amplifications are found in 206 (47.8%) of the patients [107]. Another research shows that advanced GC patient have more frequency of *PIK3CA* mutations in codon 545 than in codon 1047 [108]. A large number of researches confirm that besides NETO2, UFM1, STIL, LEMD1, SPP1 and PRL-3, miR-19a, 21, 34a, 137 and 196b, as well as lncRNA MALAT1, STXBP5-AS1 and PICART1 are involved in modulating biological functions of GC cells via PI3K/AKT pathway [109–122]. A lot of clinical trials of PI3K inhibitors (BKM120, BYL719 and GSK2636771. Table 2) and AKT inhibitors (MK2206, GSK2110183 and GDC-0068. Table 3) in GC patients try to save their lives, especially the patients with advanced or metastatic GC.

Although CRC screening has reduced the incidence and mortality nowadays [123], CRC remains one of the main reasons of tumor-related deaths worldwide (Table 1). The overall genetic alterations of PI3K/AKT pathway in CRC are observed as follows: *PIK3CA* (22%), *PIK3R1* (5%), *PIK3R2* (2.2%), *AKT1* (1.8%), *AKT2* (2.5%) and *PTEN* (8%, Table 1). Contrary to predictions, *PIK3CA* mutations do not predict aggressive clinicopathological characteristics in CRC, whereas they are closely associated with *KRAS* mutations, as well as *PIK3CA* exon 9 and 20 mutations show different tendencies with respect to *BRAF* mutation and *MSI* status [124]. Similar to ADC, the expression of PIK3IP1 is also significantly lower in CRC tissues [67]. CXCL12, NLRC3, Wnt/β-catenin target genes including BAMBI, BOP1, CKS2 and NFIL3, as well as miRNA-135b, Linc00659 and CRNDE are associated with the proliferation, invasion or metastasis of CRC cells via PI3K/AKT signaling [125–130]. As shown in Tables 2 and 3, multiple clinical trials of PI3K/AKT inhibitors in CRC patients try to yield useful inhibitors for treatment [131].

As the most common mesenchymal tumor of the digestive system, gastrointestinal stromal tumors (GISTs) mainly harbor mutually exclusive *KIT* or *PDGFRA* mutations, which lead to constitutive activation of the encoded receptor tyrosine kinase (RTK) and activation of downstream pathways including PI3K/AKT pathway [132, 133]. Genetic alterations of *PIK3CA* and *PTEN* are observed more frequency in malignant GISTs than in less malignant GISTs in 65 GIST samples with 14/65 overall genetic alterations of PI3K/AKT pathway [134]. It is noted that FASN overexpression often occurs in high-risk and metastatic GISTs, whereas combination therapy with imatinib and C75 targeting FASN has been demonstrated in vitro and vivo to down-regulate the phosphorylation levels of the KIT and PI3K/AKT/mTOR pathway [135, 136]. MiR-374b modulates proliferation and apoptosis of GIST cells through PI3K/AKT pathway [137]. Combination of imatinib mesylate (IM) and MK2206 provide obviously greater efficacy than treatment with IM or MK2206 alone in vitro and vivo preclinical study of GIST [138]. Furthermore, clinical trials of combination of Imatinib and BKM120 (NCT01468688) or BYL719 (NCT01735968, Table 2) were tested in GIST patients.

Being the third most common cause of cancer death worldwide with the mortality rate of 8.2%, hepatocellular cancer (HCC) is a distinct tumor of the digestive system and exhibits a different genetic alteration pattern of PI3K/AKT pathway, such as *PIK3CA* (3%), *PIK3R1* (1.2%), *PIK3R2* (1.5%), *AKT1* (0.7%), *AKT2* (1.1%) and *PTEN* (4%) respectively (Table 1). Similarly, PIK3IP1 also suppresses the development of HCC [67, 139]. Moreover, APLN, miR-7, -367, -1296, and -3691-5p as well as lncRNA PTTG3P and LINC01133 are associated with the proliferation, invasion, metastasis or EMT of HCC cells via PI3K/AKT pathway [140–146]. A small amount of clinical trials of PI3K inhibitors (SF1126, GSK2636771) and AKT inhibitors (MK2206) in HCC patients may give them an opportunity for relief (Tables 2 and 3).

Regarding gallbladder cancer (GBC) is the most common malignancy of the biliary tract, the general genetic abnormalities of *PIK3CA* (10%) and *PTEN* (2.3%) are found (Table 1), especially the *PIK3CA E545K* mutation rate (6.15%) [147]. Due to *ErbB2* and *ErbB3* mutations at a frequency of 7–8% in GBC, *ErbB2/ErbB3* mutation inducing PD-L1 overexpression can mediate immune escape of tumor cells via PI3K/AKT pathway in vitro [148]. In addition, EIF3d, UBR5, BRD4, TRIM31 and LINC00152 are demonstrated to contribute to cell growth or tumor metastasis of GBC cells via PI3K/AKT pathway [149–153]. Currently, only MK2206 was tested in clinical trials (NCT01859182 and NCT01425879) in GBC patients.

Pancreatic cancer (PC) is a fatal malignancy in the digestive system tumors and takes the first place among asymptomatic cancers (Table 1). Take into consideration that more than 90% of PC is pancreatic ductal adenocarcinoma (PDAC) with the 5-year overall survival (OS) rate less than 5–10% [154], novel targeting therapies are in urgent need. In contrast to the well-known genetically inactivated of *P16* (90%)*, TP53* (75%)*, DPC4* (55%), as well as activated oncogene *KRAS* (90%) and *Her2* (4–50%) in PDAC [155–159], the overall genetic aberrations of PI3K/AKT members (*PIK3CA,* 2.3% and *PTEN* 1.9%, Table 1) are less frequently. Interestingly, pancreatic cell plasticity and cancer initiation induced by *K*ras is completely dependent on wild-type p110α [160], and PAK4 interacts with p85α can affect the migration of PDAC cells [161]. Significantly, the mutations of *PIK3CG* in PDAC are also revealed [156]. EG-VEGF, TMEM158, miR-107, as well as LncRNA ABHD11-AS1, SNHG1 and AB209630 are involved in proliferation, apoptosis, metastasis or carcinogenesis of PDAC cells through PI3K/AKT pathway [162–167]. Plenty of clinical trials of PI3K inhibitors (BKM120, BYL719, GSK2636771, PKI-587, BEZ235 and LY3023414. Table 2) and AKT inhibitor (MK2206. Table 3) in PDAC patients may reveal promising therapeutic activities.

### Abnormalities of the PI3K/AKT pathway in breast and female reproductive system tumor

BC, which is Estrogen (ER)-related cancer, is the second common cancer in the world (morbidity of 11.6%) but in the first place and the most frequent cause of cancer death (mortality of 6.6%) among women worldwide (Table 1). Compared to the recognized genetically diverse of *Her2* and *TOP2A* of BCs, the overall genetic alterations of PI3K/AKT pathway are not uncommon, especially *PIK3CA* (37%) and *PTEN* (8%, Table 1). Remarkably, hotspot mutations in *PIK3CA* are frequent in ER+BCs, which account for up to 80% of BCs, and *Her2* mutations hyperactivate the HER3/PI3K/AKT/mTOR axis, leading to anti-ER resistance in ER+BCs. Hence, dual blockade of the Her2 and ER pathways is necessary for the treatment of ER+/*Her2* mutant BCs [168]. Moreover, *PIK3CA* and *MAP3K1* alterations reveal Luminal A status in ER+ metastatic BCs and the patients are likely to clinically benefit from BKM120 [169]. On the other hand, top to 70% of patients with breast cancer brain metastases (BCBM) show the activated PI3K pathway [170], and GDC-0084 induces apoptosis of *PIK3CA*-mutant BCBM cells by suppressing activation of AKT and p70 S6 kinase [171]. Additionally, PRLR/Jak2/STAT5 is the main signaling pathway for activation in mammary gland, and PRLR-triggered pro-tumorigenic pathways in BC include the PI3K/AKT pathway [172]. As well, numerous studies have shown that IRS4, CDK12, SPC24, Mfng, Transgelin 2, STX3, SOX4, PAK4, TPX2, MEG3 and miR-21, -93, -106b, -130b, -214, -361-5p, -489, -511, -564 as well as lncRNA‑HOTAIR and MALAT1 regulate tumorigenesis, proliferation, apoptosis, invasion, migration, paclitaxel resistance or anti-Her2 therapy (trastuzumab) resistance of BC cells through PI3K/AKT pathway [173–191]. And then, PI3K/AKT inhibitors have gained wide attentions, and a large number of clinical trials may have provided tremendous promises in the treatment of BC patients (shown in Tables 2 and 3).

Globally, the incidence and mortality rate of ovarian cancer (OC), which is the most frequently fatal cancer in female reproductive tract with a wide-range of pathological subtypes, are 1.6% and 1.9% respectively (Table 1). Ovarian serous cystadenocarcinoma (OSC), the leading common subtype of epithelial ovarian cancers (EOC) accounting for 90% of OC, harbors overall genetic alterations of *PIK3CA* (29%), *PIK3R1* (5%), *PIK3R2* (9%), *AKT1* (5%), *AKT2* (8%) and *PTEN* (7%, Table 1) besides the mutant *p53* in high-grade OSC (HGOSC), germline *BRCA1* and *BRCA2* mutations. Furthermore, another subtype of EOC, ovarian clear cell carcinomas (OCCCs), shows more frequently mutations of *PIK3CA* (33%) and *PTEN* (5%) in overall 97 OCCC cases, especially mutations of *PIK3CA* (46%) in the 28 cases of affinity purified OCCCs and OCCC cell lines [192], than the mutation of *PIK3CA* and *PTEN* (both < 5%) in HGOSC [193]. Huge amounts of studies have shown YAP, PKG II, SIK2, SERPIND1, miR-15b, -21, -150, -222-3p, -337-3p, -497, -503 and -936, as well as LncRNA MALAT1 and JPX modulate proliferation, apoptosis, invasion, migration, angiogenesis, progression, glucose metabolism or drug resistance of OC cells by PI3K/AKT pathway [194–207]. Some clinical trials of PI3K/AKT inhibitors or in combination with chemotherapy drugs listed in Tables 2 and 3 may help relieve the patients of OC.

Along with recent compelling evidence that OSC actually arises from the epithelial lining of fallopian tube, the true incidence of primary fallopian tube carcinoma (PFTC) has been substantially underestimated, which was previously considered as a rare neoplasm accounting for 0.14–1.8% of genital malignancies [208, 209]. Furthermore, aberrant p53/KRASV12/c-Myc or p53/KRASV12/PI3K/AKT signaling is the minimum requirement for fallopian tube secretory epithelial cells (FTSECs) carcinogenesis [210], and increased copy number of *PIK3CA* has been observed in six fallopian tube carcinomas (FTCs) [211]. Thus, although the studies of PI3K/AKT signaling in FTC are numbered, there are still several clinical trials of PI3K/AKT inhibitors trying to treat patients with FTCs (Tables 2 and 3).

Cervical cancer (CC) is a prominent example of HPV-related cancer, accounting for 3.2% of all human cancers with the mortality rate of 3.3% (Table 1). A litany of genetic alterations induced by HPVs in CC activate four major upstream pathways (GFR, Notch receptor, RAS isoforms and p110α) to stimulate host cell survival, proliferation and carcinogenesis through the PI3K/AKT/mTOR pathway. Considerable overall genetic alterations of PI3K/AKT pathway in CC have emerged with *PIK3CA* (39%) and *PTEN* (13%, Table 1). In particular, the mutations of *PIK3CA E542K* and *E545K* promote glycolysis and proliferation of CC in vitro and vivo [212]. NBPF1, ARHGAP17, miR-99b, -181a2/181b2, -338, -383, -433 and -489, as well as LncRNA ANRIL, CRNDE, NEAT1 and LINC01305 are involved in the proliferation, invasion, autophagy or EMT via PI3K/AKT pathway [213–224]. Currently, only preclinical trials of PI3K inhibitor LY294002 has revealed it significantly radiosensitized CC cell lines in vitro and vivo [225, 226], and the terminated clinical trials of AKT inhibitor GSK2141795 (NCT01958112, Table 3) has tried to display a novel treatment approach to patients of CC.

Attributed to the global incidence (2.1%) and mortality rate (0.94%) of corpus uteri cancer, which is usually referred to endometrial cancer (EC), EC researches have gained a big momentum in recent years. Particularly, the endometrioid type of EC (EEC) progressing from intraepithelial endometrial neoplasia in a large proportion of cases belongs to ER-related cancer, and is directly associated with inactivation of PTEN. Hereby, the remarkable overall genetic alterations of PI3K/AKT pathway are shown in EC, such as: *PIK3CA* (34%), *PIK3R1* (19%), *PIK3R2* (5%), *AKT1* (3%) and *AKT2* (5%), especially *PTEN* (32%, Table 1). What’s more, it’s revealed that the majority of the G3 EEC samples have exhibited *PIK3CA* mutations (39%) and *PTEN* mutations (67%) [227]. Moreover, JQ1, NEDD4, PDCD4, miR-101, -494-3p, Lnc RNA LINP1 and MEG3 have shown their aptitudes for controlling tumorigenesis, proliferation, apoptosis, invasion, progression of EC cells via PI3K/AKT pathway [228–234]. Thus, EC patients may get benefit from the mounting clinical trials of PI3K/AKT inhibitors listed in Tables 2 and 3.

### Dysregulation of the PI3K/AKT Pathway in the genitourinary system tumors

The morbidity of PCa ranks third in the world (7.1%) since men obtain a small but finite benefit from PCa screening in terms of PCa-specific mortality, which is estimated as 3.8% globally [235] (Table 1). Seeing that loss of function of PTEN, resulting in dysregulated activation of the PI3K signaling network, is recognized as one of the most common driving events in PCa development [236], the overall genetic alterations of PI3K/AKT pathway in PCa have demonstrated with *PIK3CA* (6%), and visible *PTEN* (18%, Table 1). Sexual hormones have been historically associated with PCa for the androgen deprivation therapy (ADT), but scientific evidences including the increasingly emerging of castration resistant prostate cancer (CRPC) are inconsistent to decide whether their involvement is aetiological or a phenotype component of the disease. However, similar to BC, PRLR/Jak2/STAT5 is also the main signaling pathway for activation in prostate gland, and PRLR-triggered pro-tumorigenic pathways in PCa include PI3K/AKT [172]. In addition, AEP, SCL/TAL1, SIRT3, Snail, MED15, STIM1, ST6Gal-I, Glyoxalase 2, ASF1B, GPCR48/LGR4, AP4, GCN5, SAG/RBX2 E3, miR-7, -101, -129, -133a-3p, and -4638-5p, as well as LncRNA HCG11 and ATB govern tumorigenesis, progression, metastasis, EMT or castration resistant of PCa cells via PI3K/AKT pathway [237–256]. Preclinical trial of dual BRD4/PI3K inhibitor SF2523 [257] as well as a few of clinical trials of PI3K/AKT inhibitors may develop new therapeutic strategies for PCa patients (Tables 2 and 3).

Kidney cancer (KC) is a malignancy originating in the urinary tubular epithelial system of the renal parenchyma, which mainly means renal cell carcinoma (RCC). Accompanying with the recent hunt for the genetics causes of KC, such as *TFE3, TFEB,* or *MITF *gene fusions, the overall genetic alterations of PI3K/AKT pathway comprising *PIK3CA* (2.8%), *PIK3R1* (0.4%), *PIK3R2* (0.3%), *AKT1* (0.5%), *AKT2* (0.6%) and *PTEN* (4%, Table 1) are captured in KC. To a further extent, PI3K/AKT/mTOR is identified as a highly enriched pathway in translocation RCC with *TFE3* fusion (*TFE3*-tRCC) by miRNA microarray analysis [258]. Besides that PIK3R1 regulates EMT and stem-like phenotype of RCC cells through the AKT/GSK3β/CTNNB1 pathway [259], FoxO, PKCε, TPD52, NOTCH1, ETS2, miR-19b, -122, -182, -193a-3p, -195, and -224, as well as LncRNA MALAT1, TP73-AS1 and HOTTIP modulate proliferation, apoptosis, invasion, metastasis, or EMT via PI3K/AKT pathway [260–272]. However, only a few of clinical trials of PI3K/AKT inhibitors in touch try to offer hope for KC patients (Tables 2 and 3).

Bladder cancer (BLCA) is complex disease mainly consisting of non-muscle-invasive bladder cancer (NMIBC, about 70%), and muscle-invasive and metastatic bladder cancer (MIBC, about 30%). Indeed, *PIK3CA* mutations are considered as an early genetic alteration associated with *FGFR3* mutations in superficial papillary NMIBC [273] and the activation of the PI3K/AKT pathway is identified to induce urothelial carcinoma of the renal pelvis [274]. And the overall genetic alterations of *PIK3CA* (24%) are described in BLCA (Table 1). It is further shown that PPARγ, Sema4D, CCDC34, miR-29c, -143, -145 and -294, as well as LncRNA ATB, LINC00641, HULC, DUXAP10 and UCA1 regulate proliferation, migration, or invasion of BLCA cells via PI3K/AKT pathway [275–285]. Even though there is a significant unmet need for new therapies, however, at present only a small amount of clinical trials of BKM120 and GSK2636771 try to find out what PI3K inhibitor’s prospects bring to the BLCA patients (Table 2).

Testicular cancer (Te Ca) is the most common malignancy among men between 14 and 44 years in the world. Testicular germ cell tumors (TGCTs) are classified as seminoma and non-seminoma. Among the numerous genetic and environmental factors, cryptorchidism is the most common risk factor. Compared to the noted *KRAS* and *NRAS* mutations in TGCTs [286], the overall genetic alterations frequency of *PIK3CA* (3%), *PIK3R1* (1.3%), *AKT1* (0.7%) and *PTEN* (0.7%) are much less, even though mutations in *PIK3CA* and *AKT1* are observed exclusively in cisplatin-resistant TGCTs [287]. AXIN1, TDRG1 and LncRNA H19 regulate cell viability, apoptosis or cisplatin resistance via the PI3K/AKT/mTOR signaling pathway [288–290]. Unfortunately, PI3K/AKT inhibitors have not yet applied in clinical trials of TGCT patients up to now.

### Description of the PI3K/AKT pathway in the hemato-immune system tumors

Hematologic cancers are associated with hemato-immune system, which comprise lymphomas, myelomas and leukemias. Lymphoma, which is classified with Hodgkin's lymphoma (HL) and non-Hodgkin's lymphoma (NHL), and multiple myeloma (MM) emanate from the cells of the immune system, while leukemia originates from blood-forming tissues such as the bone marrow [291, 292].

HL is a rare B-cell malignant neoplasm approximately accounting for 0.44% of all new cancers annually, which is classified into two discrete disease entities: classical Hodgkin lymphoma (CHL) and nodular lymphocyte-predominant Hodgkin lymphoma (NLPHL). With four subgroups including nodular sclerosis (NSCHL), mixed cellularity (MCCHL), lymphocyte depletion (LDCHL), and lymphocyte-rich (LRCHL), CHL is relatively less known about genetic lesions owing to the fact that the neoplastic Hodgkin- and Reed-Sternberg (HRS) cells constituting only a small proportion of the tumor tissue [293]. But the prevalence of EBV in HRS cells varies according to the histological subtype and epidemiologic factors from highest frequency in MCCHL to the lowest in NSCHL, and EBV-encoded LMP1 utilizes the PI3K/AKT/mTOR signaling axis to induce ectopic CD137 expression in HRS cells, which results in enhancing the proliferation rate of HRS cells [294, 295]. Furthermore, differences related to EBV status or histological subtypes are observed for PI3K signaling in pediatric HL patients by using hybrid capture-targeted next-generation sequencing of circulating cell-free DNA (ccfDNA), where MCCHL and EBV+ cases were less frequently affected by mutations in *ITPKB* and *GNA13* genes [296]. Recent evidences revealing that germinal center B-cells (GCB cells) are the cellular origin of HRS cells [294], and the facts that PRMT5 is upregulated by B-cell receptor signaling and forms a positive-feedback loop with PI3K/AKT in both activated B cell-like (ABC) and GCB cells of diffuse large B cell lymphoma (DLBCL) [297] suggest that PI3K/AKT may promote lymphomagenesis of GCB cells in HL, which is a remarkable coincidence with the other evidences that the PI3K/AKT pathway plays a pathogenetic role in HL [298, 299]. Thus, novel therapeutic options targeted PI3K/AKT pathway promote apoptosis or cell death, as well as regulate tumor microenvironment (TME) of HL cells in preclinical studies [300–302], and patients may get beneficial strategy in clinical trials of PI3K/AKT inhibitors (Tables 2 and 3).

As the most common malignancies of hemato-immune system in the world, NHL represents a wide spectrum of illnesses that vary from the most indolent to the most aggressive malignancies, which encompasses 2 main type: mature B-cell neoplasms (B-NHL, 85–90%) and mature T-cell and natural killer (NK)-cell neoplasms (T/NK-NHL, 10–15%; 2016 WHO). Indolent B-cell lymphomas (iB-NHL) represents 35–40% of NHL, and the most common subtypes include follicular lymphoma (FL), chronic lymphocytic leukemia/small lymphocytic lymphoma (CLL/SLL), a fraction of mantle cell lymphoma (MCL) cases, extramedullary, nodal and splenic marginal zone lymphoma (MZL), and lymphoplasmacytic lymphoma (LPL). On the other hand, the most common subtypes of aggressive B-NHL are large B-cell lymphomas, which is composed of DLBCL, not otherwise specified (NOS, 80%) and additional 13 specific variants of DLBCL (20%) including anaplastic (ALK + LBCL) and primary mediastinal lymphoma (PMLBCL), and other various kinds of DLBCL [303–306]. Anyway, the overall genetic alterations of *PIK3CA* (0.4%), *PIK3R1* (0.5%), *PIK3R2* (0.1%), *AKT1* (0.1%), *AKT2* (0.1%) and *PTEN* (1.1%, Table 1) are observed statistically in NHL.

Apparently, two specific lymphomas, FL and DLBCL, account for about 65% of all NHL, and more importantly, the genomic profile of transformed FL shares similarities with that of GCB de novo DLBCL, and thus a thorough knowledge of these two entities related with PI3K/AKT pathway is essential [307–309]. Despite the recognized fact that overwhelming majority of FL cases have the characteristic (14;18) translocation involving the IgH/bcl-2 genes, while B-cells "arrested" in germinal centers of FL acquire dozens of additional genetic aberrations that influence key pathways controlling their physiological development including B Cell Receptor (BCR) signaling, PI3K/AKT pathway, and so on [310, 311]. Especially, the facts that deletion of *PIK3CD* results in decreased number of marginal zone (MZ) B cells and pleural/peritoneal cavities in mice, as well as the evidences that *PIK3CD*-depleted B cells also fail to proliferate in vitro in response to BCR or CD40 signals and have impaired both humoral T-cell-dependent and T-cell-independent responses suggest that p110δ plays a critical role in B cell homeostasis and function [312–314]. Consequently, following with the world's first selective PI3Kδ inhibitor CAL-101 was approved by the FDA for the treatment of FL, CLL and SLL in 2014 [315] [NCT01282424, NCT02136511], the PI3K/AKT inhibitors have shown remarkable activity in an increasing subset of patients with NHL [316] (Tables 2, 3). Copanlisib (BAY 80-6946) and Duvelisib (IPI-145) are newly approved PI3K inhibitors that offer objective, although relatively short-lasting, responses in patients with heavily pre-treated FL and other NHL, and more such targeted agents may be approved soon [307, 317–320] (Tables 2 and 3).

As aforementioned, DLBCL is a highly aggressive heterogeneous disease with two subtypes: GCB and ABC [297]. One study shows that deregulation of the PI3K/AKT pathway by the inactivation of PTEN are found in 55% of GCB-DLBCL cases, but only in 14% of non-GCB-DLBCL and worsens prognosis in 248 primary DLBCL patients [308]. Another study finds the *PIK3CA* amplification of 12.7% and PTEN loss of 12.2% in DLBCL [321]. Furthermore, upregulation of PRMT5 and CXCR4 are involved in lymphomagenesis or resistance mechanism via the PI3K/AKT pathway in DLBCL cells [297, 322]. Preclinical trial of BAY80-6946 in DLBCL cells [323] and the clinical trials of BAY80-6946, INCB050465, CUDC-907 and MK2206 in patients with DLBCL have improved our ability to manage patients with this disorder (Table 2).

T/NK-NHL is a heterogeneous group of malignancies often associated with poor clinical outcomes, and each malignancy within this group is characterized by unique clinicopathologic features, while T cell receptor/NF/kB (TCR/NF/kB) signaling highly enriched and dysregulation of JAK/STAT pathway, specifically aberrant STAT3 activation, are the common feature among these lymphomas [324–326]. A study with 426 adult T cell leukemia/lymphoma (ATL) cases associated with human T cell leukemia virus type-1 (HTLV-1) infection shows that *PI3KCD* mutation is also observed in 9 of 370 (2.4%) cases besides the highly enriched for TCR/NF/kB signaling, T cell trafficking and other T cell-related pathways [324]. In addition, the alterations of PI3K signaling are involved in the multilobulated nucleus formation and cell proliferation in ATL cells [327]. Therefore, preclinical trial of CAL-101 inducing apoptosis in ATL cells [328] and a series of clinical trials of PI3K/AKT inhibitors are expected to offer new treatment regimens for patients with T/NK-NHL [316] (Tables 2, 3).

MM accounts for 0.88% of all cancers with the mortality rate (1.1%). Almost all MM patients evolve from an asymptomatic pre-malignant stage termed monoclonal gammopathy of undetermined significance (MGUS). Despite that hotspot mutations of *PIK3CA* (E542K, E545K and H1047R) and *AKT1* genes (E17K) are absent in MM [329], the R310C mutation of *PIK3CA* gene [330] is identified in some cases of MM, as well as ROR2 drives the interaction of MM cells with TME through AKT activation [331]. Furthermore, only the blockade of *PIK3CA* is sufficient to induce cell death in a sizeable subgroup of MM samples, and *PIK3CA* inhibitor BYL-719 in combination treatments with other compounds establishes anti-myeloma agents resulted in strongly enhanced MM cell death [332]. Therefore, some preclinical studies have examined PI3K/AKT pathway inhibitors in MM, such as TAS-117, PI-103 and BEZ235 [333–335]. Fortunately, some of the clinical trials of PI3K/AKT inhibitors have demonstrated encouraging clinical activity in relapsed and relapsed/refractory (R/R) MM [336–339] (NCT01002248; NCT01476137; NCT00881946) (Tables 2 and 3).

The definition of leukemia is increasingly employed that an aberrant hyper-proliferation of immature blood cells either of the myeloid or lymphoid lineages forms liquid cancer, which is classified with acute or chronic. With morbidity (2.4%) and mortality rate (3.2%) across the world (Table 1), leukemia is a series of life-threatening malignant diseases, particularly in the adolescent and young adult (AYA) population, in which the acute leukemias are most prevalent [340]. Apart from the iconic *BCR/ABL* oncogene formation in chronic myeloid leukemia (CML) and the genetic abnormalities frequently linked to treatment resistance and poor patient outcome in acute myeloid leukemia (AML), for example the unique *PML-RARA* fusion in acute promyelocytic leukaemia (APL; AML M3), the PI3K/AKT pathway can function as a prosurvival factor in leukemia stem cells and early committed leukemic precursors with the following facts: Firstly, the overall genetic alterations of *PIK3CA* (0.6%), *PIK3R1* (0.6%), *PIK3R2* (0.4%), *AKT1* (0.5%), *AKT2* (0.1%) and *PTEN* (0.7%, Table 1) are observed in leukemia (Table 1). Secondly, PTEN plays critical roles in regulating not only hematopoietic stem cell activity through a Niche-dependent mechanism, but also hematopoiesis and leukemogenesis [341–343]. Furthermore, TAL1, c-Jun, EZH2, TRIM22, ETV6/RUNX1, miR-7, -22, -26b, -103, -125b, -126, -139-5p, -181c, -193a, -628, and -3142, as well as LncRNA HULC, UCA1, linc00239 and LINC00265 control leukemogenesis, proliferation, apoptosis or chemoresistance via PI3K/AKT pathway [344–363]. Hereafter, PI3K/AKT pathway inhibition is regarded as a therapeutic approach [364, 365] followed by the preclinical studies in leukemia cells [366, 367] in spite of the upregulated expression of P2RY14 in acute leukemia cells resistant to PI3K/mTOR inhibition [368]. Since CAL-101 has been approved for marketing in patients with CLL/SLL, the clinical trials of PI3K/AKT inhibitors such as: BAY80-6946, KM120, YY-20394, BEZ235, PKI-587, IPI-145, CAL-101, TGR-1202, MK2206 and GSK2141795 try to seek new therapeutic approach in relapse or refractory patients with CLL or newly diagnosed AML and acute lymphocytic leukemia (ALL, Tables 2 and 3).

### Featuring the PI3K/AKT pathway in the bone and soft tissue tumors

Osteosarcoma (OS) is the most frequent primary solid malignancy of bone with the presence of malignant mesenchymal cells which produce osteoid and/or immature bone. The incidence of OS is higher in adolescence (8–11/million/year) than in the general population (2–3/million/year), and > 90% of OS patients died from pulmonary metastases before polychemotherapy. Although the biological and genetic studies of OS have made substantial progress, there has been no qualitative breakthrough in treatment over the past 30 years. Besides the alterations of *TP53*, *RB1*, ATRX and DLG2 in OS, total genetic alterations in the PI3K/AKT/mTOR pathway are observed in 14 of 59 (24%) OS patients, and *PIK3CA* and *mTOR* are vital for the proliferation and survival of OS cells [369] (Table 1). Furthermore, dual PI3K/mTOR inhibitors are effective at inducing apoptosis in primary OS cell cultures in vitro in both human and mouse OS, while specific PI3K or mTOR inhibitors are not effective [370], which is consistent with the preclinical study’s result that BEZ235 inhibits proliferation and tumor development of OS cells in vivo [371].

Ewing's sarcoma (EWS), the second most common bone tumor in children and adolescents, is identified by the characteristic t (11;22) chromosomal translocation and resulting oncogenic *EWS–FLI1* fusion, for which no cure is currently available. Overall genetic alterations of the PI3K/AKT pathway are observed in EWS cases with *PIK3CA* (1.4%), *PIK3R1* (0.5%) and *PTEN* (0.5%, Table 1), which play an important role in EWS pathogenesis [372]. Moreover, SOX2, Ski, miR-30d and -185 regulate proliferation, apoptosis, migration or progression of EWS cells though PI3K/AKT pathway [373–376]. In addition, hnRNPM motifs are significantly enriched under the inhibition of the PI3K/AKT/mTOR pathway by BEZ235 in EWS cells. On the other hand, hnRNPM down-expression revokes the BEZ235-induced splicing changes including hnRNPM binding sites, enhanced BEZ235 cytotoxicity and limited the clonogenicity of EWS cells [377].

Currently, pediatric patients of OS or EWS may be beneficial from the ongoing clinical trials of BAY80-6946 (NCT03458728) and LY3023414 (NCT03213678, Table 2).

### The trait of the PI3K/AKT pathway in skin cancer

Skin cancer is the most common carcinoma, affecting millions worldwide annually, which generally divided into malignant melanoma and non-melanoma skin cancer. Cutaneous melanoma ranks 20th among most common cancers worldwide and rapidly becomes life-threatening once it has spread. Even though solar ultraviolet exposure is the main environmental risk factor for cutaneous melanoma development, there are still genetic susceptibility factors, such as germline mutations in *p16* or *CDK4*, and genesis of melanoma, such as the main genetic drivers *BRAF*, *NF1* and *NRAS* mutations [378, 379]. Since *BRAF*^*V600E*^*-*mutated melanomagenesis is often accompanied by silencing of PTEN [380], the increasing genetic alterations in PI3K/AKT pathway have been observed in melanoma including: *PIK3CA* (5%) and *PTEN* (12%, Table 1). Notably, dysfunction mutations of NF1 induce BRAF inhibitor resistance by activating RAS and its downstreams including both MAPK and PI3K/AKT/mTOR pathways in cutaneous melanoma [381, 382]. Even more, the onset of MEK1/2 inhibitor resistance in *BRAF*-mutated melanoma can be forestalled by PI3K blockade [383]. Other than that, ROR1, FOXC1, MIF, TGFβ, lncRNA SNHG17, MIAT, MHENCR, OR3A4 and H19 regulate proliferation, progression, migration, invasion, metastasis or EMT-like transition though PI3K/AKT pathway in melanoma cells [384–392]. And now, a limited number of clinical trials of PI3K/AKT pathway inhibitors (BKM120, PX-866, GSK2636771, GSK2141795 and MK2206) try to find new ways other than current classic RAF/MEK/MAPK pathway inhibitors to treat the patients with metastatic or advanced melanomas (Tables 2 and 3).

## Points of dispute or unanswered questions

**Fig. 1:**
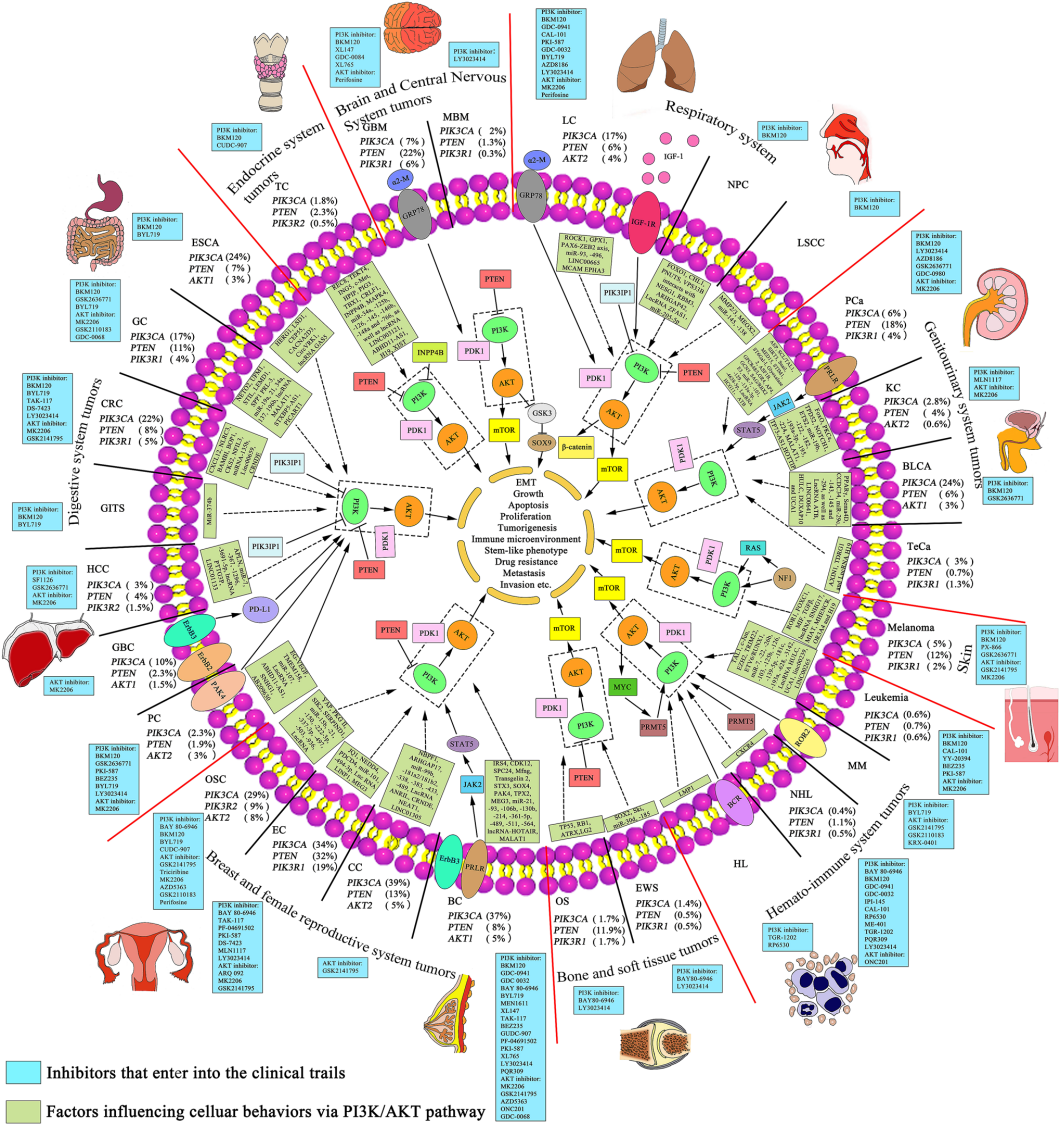
Overview of the PI3K/AKT signaling cascades in cancers

In general, ATC, NSCLC, EC, GC, CRC, BC, OC, CC, EC and BLCA exhibit higher frequencies of *PIK3CA* mutations than other tumors, while *PTEN* mutations are predominantly found in GBM, EC and PCa (Fig. 1, Table 1). No matter what kind of the genetic alteration happens in PI3K/AKT pathway, or the factor influences cellular behaviors via PI3K/AKT pathway, it leads to the hyper-activation of PI3K/AKT pathway. Growing evidences have shown that the hyper-activation of PI3K/AKT pathway in malignant tumor influences the tumorigenesis, proliferation, growth, apoptosis, invasion, metastasis, EMT, stem-like phenotype, immune microenvironment, drug resistance of tumor cells (Fig. 1). Interestingly, some protein may play a dual role in PI3K/AKT pathway. For instance, unlike the previous understanding that INPP4B is a negative regulator of PI3K/AKT pathway in TC cells in vivo [49], the tumor-promoting features of INPP4B have yet been observed in leukemia and BC [393–395]. Why and how the INPP4B is a double-edged sword in PI3K/AKT pathway is still a puzzle and it needs further research to evaluate the evidences.

## Potential research/future

More and more promising PI3K/AKT pathway inhibitors seem to be useful to overcome malignant tumor, especially CAL-101 treated in patients with hemato-immune system tumors has achieved exhilarating results. Obviously, CAL-101 not only causes a rapid and sustained reduction in lymphadenopathy, but also regulates the immune environment in CLL [396, 397]. However, things are more complicated than our envisage and there is always coexist with abnormal activity of other pathways interacted with PI3K/AKT pathway in tumors. For example, AKT inhibition induces the expression and phosphorylation of multiple RTKs, and the activated RTK signaling may attenuate their antitumor activity in BC cells, which suggest that combined inhibition of AKT and HER kinase activity is more effective than either alone [398]. There are some other embarrassments findings that small molecule PI3K/AKT pathway inhibitors could promote the (re)phosphorylation of AKT2 which is linked to the redistribution and adaptive reprogramming of mitochondria, contributing to drug resistance and metastasis in GBM cells [399, 400]. Thence, novel combination therapies that target mitochondrial adaptation and PI3K pathway may achieve better efficacies than either alone in the clinic.

Collectively, we hope to feature PI3K/AKT pathway in cancers to the clinic and bring the promise of the novel inhibitors to the patients for targeted therapies.

## Abbreviations

ABC: Activated B cell-like
AI: Aromatase inhibitor
ALL: Acute lymphoblastic leukemia
AML: Acute myeloid leukemia
AKT: Protein kinase B
ATC: Anaplastic thyroid cancer
ATL: Adult T cell leukemia/lymphoma
AYA: Adolescent and young adult
BC: Breast cancer
BCBM: Breast cancer brain metastases
BCL: B-cell lymphoma
BTKi: BTK inhibitors
ccfDNA: Circulating cell-free DNA
CHL: Classical Hodgkin lymphoma
CLL/SLL: Chronic lymphocytic leukemia or small lymphocytic lymphoma
CRC: Colorectal carcinoma
CRPC: Castration resistant prostate cancer
CSC: Cancer stem cell
EBV: Epstein–Barr virus
EC: Endometrial cancer
EEC: Endometrioid type of EC
EMT: Epithelial–mesenchymal transition
EOC: Epithelial ovarian cancers
ER: Estrogen
ESCA: Esophageal carcinoma
ESCC: Esophageal squamous cell carcinoma
EWS: Ewing's sarcoma
FL: Follicular lymphoma
FTC: Fallopian tube carcinoma
GBM: Glioblastoma multiforme
GC: Gastric cancer
GCB cells: Germinal center B-cells
GCO: Global Cancer Observatory
GIST: Gastrointestinal stromal tumor
HCC: Hepatocellular carcinoma
HGOSC: High-grade OSC
HL: Hodgkin's lymphoma
HNSCC: Head-and-neck squamous cell carcinoma
HR: Hormone receptor
HRS cells: Hodgkin- and Reed-Sternberg cells
HSCT: Hematopoietic stem cell transplant
iB-NHL: Indolent B cell non-Hodgkin's lymphoma
iNHL: Indolent non-Hodgkin's lymphoma
IS: Isoform-selective
IV: Intravenous
LPL: Lymphoplasmacytic lymphoma
MBC: Metastatic breast cancer
MBM: Medulloblastoma
mTOR: Mammalian target of rapamycin
MCL: Mantle cell lymphoma
MM: Multiple myeloma
MRD: Maximum recommended dose
MTD: Maximum tolerated dose
mTORi: Rapamycin inhibitor
MZL: Marginal zone lymphoma
NHL: Non-Hodgkin's lymphoma
NK/TCL: NK/T cell lymphomas
NLPHL: Nodular lymphocyte-predominant Hodgkin lymphoma
NSCLC: Non-small cell lung cancer
OC: Ovarian cancer
ORR: Objective response rate
OS: Osteosarcoma
OSC: Ovarian serous cystadenocarcinoma
PBR: Placebo in combination with bendamustine and rituximab
PC: Pancreatic cancer
PCa: Prostate cancer
PCNSL: Primary central nervous system lymphoma
PD: Pharmacodynamics
PDAC: Pancreatic ductal adenocarcinoma
PDTC: Poorly differentiated thyroid cancer
PFS: Progression-free survival
PI3K: Phosphatidylinositol 3-kinase
PK: Pharmacokinetics
PNETs: Pancreatic neuroendocrine neoplasms
PO: Pharmacokinetics of oral
PTC: Papillary thyroid cancer
PTCL: Peripheral T-cell lymphoma
RCC: Renal cell cancer
RP2D: Recommended phase 2 dose
SCCHN: Squamous cell carcinoma of the head and neck
SCLC: Small cell lung cancer
SE: Side effects
SEC: Serous type of EC
TC: Thyroid cancers
TCL: T-cell lymphoma
TME: Tumor microenvironment
TNBC: Triple negative breast cancer
UM: Uveal melanoma
XRT: Radiation therapy

## References

[1] Zhou M, Wang H, Zeng X (2019). Mortality, morbidity, and risk factors in China and its provinces, 1990–2017: a systematic analysis for the Global Burden of Disease Study 2017. Lancet.

[2] Fayard E, Moncayo G, Hemmings BA (2010). Phosphatidylinositol 3-kinase signaling in thymocytes: the need for stringent control. Sci Signal.

[3] Limon JJ, Fruman DA (2012). Akt and mTOR in B cell activation and differentiation. Front Immunol.

[4] Yuan TL, Cantley LC (2008). PI3K pathway alterations in cancer: variations on a theme. Oncogene.

[5] Marshall JDS, Whitecross DE, Mellor P (2019). Impact of p85alpha alterations in cancer. Biomolecules.

[6] Carmona FJ, Montemurro F, Kannan S (2016). AKT signaling in ERBB2-amplified breast cancer. Pharmacol Ther.

[7] Alessi DR, Andjelkovic M, Caudwell B (1996). Mechanism of activation of protein kinase B by insulin and IGF-1. Embo J.

[8] Sarbassov DD, Guertin DA, Ali SM (2005). Phosphorylation and regulation of Akt/PKB by the rictor-mTOR complex. Science.

[9] Walker KS, Deak M, Paterson A (1998). Activation of protein kinase B beta and gamma isoforms by insulin in vivo and by 3-phosphoinositide-dependent protein kinase-1 in vitro: comparison with protein kinase B alpha. Biochem J.

[10] Meier R, Alessi DR, Cron P (1997). Mitogenic activation, phosphorylation, and nuclear translocation of protein kinase Bbeta. J Biol Chem.

[11] Franke TF, Yang SI, Chan TO (1995). The protein kinase encoded by the Akt proto-oncogene is a target of the PDGF-activated phosphatidylinositol 3-kinase. Cell.

[12] Recabarren D, Alarcon M (2017). Gene networks in neurodegenerative disorders. Life Sci.

[13] Yu J, Zhang Y, McIlroy J (1998). Regulation of the p85/p110 phosphatidylinositol 3'-kinase: stabilization and inhibition of the p110alpha catalytic subunit by the p85 regulatory subunit. Mol Cell Biol.

[14] Chagpar RB, Links PH, Pastor MC (2010). Direct positive regulation of PTEN by the p85 subunit of phosphatidylinositol 3-kinase. Proc Natl Acad Sci USA.

[15] Brown JS, Banerji U (2017). Maximising the potential of AKT inhibitors as anti-cancer treatments. Pharmacol Ther.

[16] Huck BR, Mochalkin I (2017). Recent progress towards clinically relevant ATP-competitive Akt inhibitors. Bioorg Med Chem Lett.

[17] Dolecek TA, Propp JM, Stroup NE (2012). CBTRUS statistical report: primary brain and central nervous system tumors diagnosed in the United States in 2005–2009. Neuro Oncol.

[18] Zhao HF, Wang J, Shao W (2017). Recent advances in the use of PI3K inhibitors for glioblastoma multiforme: current preclinical and clinical development. Mol Cancer.

[19] Brennan CW, Verhaak RG, McKenna A (2013). The somatic genomic landscape of glioblastoma. Cell.

[20] Weber GL, Parat MO, Binder ZA (2011). Abrogation of PIK3CA or PIK3R1 reduces proliferation, migration, and invasion in glioblastoma multiforme cells. Oncotarget.

[21] Zhao HF, Wang J, Jiang HR (2016). PI3K p110beta isoform synergizes with JNK in the regulation of glioblastoma cell proliferation and migration through Akt and FAK inhibition. J Exp Clin Cancer Res.

[22] Luk SK, Piekorz RP, Nurnberg B (2012). The catalytic phosphoinositol 3-kinase isoform p110delta is required for glioma cell migration and invasion. Eur J Cancer.

[23] Chen H, Mei L, Zhou L (2011). PTEN restoration and PIK3CB knockdown synergistically suppress glioblastoma growth in vitro and in xenografts. J Neurooncol.

[24] Jamieson S, Flanagan JU, Kolekar S (2011). A drug targeting only p110alpha can block phosphoinositide 3-kinase signalling and tumour growth in certain cell types. Biochem J.

[25] Holand K, Boller D, Hagel C (2014). Targeting class IA PI3K isoforms selectively impairs cell growth, survival, and migration in glioblastoma. PLoS ONE.

[26] Jones NM, Rowe MR, Shepherd PR (2016). Targeted inhibition of dominant PI3-kinase catalytic isoforms increase expression of stem cell genes in glioblastoma cancer stem cell models. Int J Oncol.

[27] Joy A, Kapoor M, Georges J (2016). The role of AKT isoforms in glioblastoma: AKT3 delays tumor progression. J Neurooncol.

[28] Kaley TJ, Panageas KS, Mellinghoff IK (2019). Phase II trial of an AKT inhibitor (perifosine) for recurrent glioblastoma. J Neurooncol.

[29] Alvarez-Garcia V, Tawil Y, Wise HM (2019). Mechanisms of PTEN loss in cancer: it's all about diversity. Semin Cancer Biol.

[30] Verhaak RG, Hoadley KA, Purdom E (2010). Integrated genomic analysis identifies clinically relevant subtypes of glioblastoma characterized by abnormalities in PDGFRA, IDH1, EGFR, and NF1. Cancer Cell.

[31] Dadey DYA, Kapoor V, Hoye K (2017). Antibody targeting GRP78 enhances the efficacy of radiation therapy in human glioblastoma and non-small cell lung cancer cell lines and tumor models. Clin Cancer Res.

[32] Li HF, Kim JS, Waldman T (2009). Radiation-induced Akt activation modulates radioresistance in human glioblastoma cells. Radiat Oncol.

[33] Misra UK, Pizzo SV (2014). Activated alpha2-macroglobulin binding to cell surface GRP78 induces T-loop phosphorylation of Akt1 by PDK1 in association with Raptor. PLoS ONE.

[34] Gottardo NG, Gajjar A (2006). Current therapy for medulloblastoma. Curr Treat Options Neurol.

[35] Suryo Rahmanto A, Savov V, Brunner A (2016). FBW7 suppression leads to SOX9 stabilization and increased malignancy in medulloblastoma. Embo J.

[36] Tan Y, Sangfelt O, Spruck C (2008). The Fbxw7/hCdc4 tumor suppressor in human cancer. Cancer Lett.

[37] Wang Z, Inuzuka H, Zhong J (2012). Tumor suppressor functions of FBW7 in cancer development and progression. FEBS Lett.

[38] Xing M (2010). Genetic alterations in the phosphatidylinositol-3 kinase/Akt pathway in thyroid cancer. Thyroid.

[39] Nilubol N, Kebebew E (2015). Should small papillary thyroid cancer be observed? A population-based study. Cancer.

[40] Landa I, Ibrahimpasic T, Boucai L (2016). Genomic and transcriptomic hallmarks of poorly differentiated and anaplastic thyroid cancers. J Clin Invest.

[41] Liu D, Shen X, Zhu G (2015). REC8 is a novel tumor suppressor gene epigenetically robustly targeted by the PI3K pathway in thyroid cancer. Oncotarget.

[42] Zheng Z, Zhou X, Cai Y (2018). TEKT4 promotes papillary thyroid cancer cell proliferation, colony formation, and metastasis through activating PI3K/Akt pathway. Endocr Pathol.

[43] Gao W, Han J (2018). Overexpression of ING5 inhibits HGF-induced proliferation, invasion and EMT in thyroid cancer cells via regulation of the c-Met/PI3K/Akt signaling pathway. Biomed Pharmacother.

[44] Byeon HK, Na HJ, Yang YJ (2016). c-Met-mediated reactivation of PI3K/AKT signaling contributes to insensitivity of BRAF(V600E) mutant thyroid cancer to BRAF inhibition. Mol Carcinog.

[45] Wang SC, Chai DS, Chen CB (2015). HPIP promotes thyroid cancer cell growth, migration and EMT through activating PI3K/AKT signaling pathway. Biomed Pharmacother.

[46] Xu J, Cai J, Jin X (2015). PIG3 plays an oncogenic role in papillary thyroid cancer by activating the PI3K/AKT/PTEN pathway. Oncol Rep.

[47] Wang N, Li Y, Wei J (2019). TBX1 functions as a tumor suppressor in thyroid cancer through inhibiting the activities of the PI3K/AKT and MAPK/ERK pathways. Thyroid.

[48] Yu ST, Zhong Q, Chen RH (2018). CRLF1 promotes malignant phenotypes of papillary thyroid carcinoma by activating the MAPK/ERK and PI3K/AKT pathways. Cell Death Dis.

[49] Li Chew C, Lunardi A, Gulluni F (2015). In vivo role of INPP4B in tumor and metastasis suppression through regulation of PI3K-AKT signaling at endosomes. Cancer Discov.

[50] Wang W, Shen T, Dong B (2019). MAPK4 overexpression promotes tumor progression via noncanonical activation of AKT/mTOR signaling. J Clin Invest.

[51] Ma Y, Qin H, Cui Y (2013). MiR-34a targets GAS1 to promote cell proliferation and inhibit apoptosis in papillary thyroid carcinoma via PI3K/Akt/Bad pathway. Biochem Biophys Res Commun.

[52] Bu Q, You F, Pan G (2017). MiR-125b inhibits anaplastic thyroid cancer cell migration and invasion by targeting PIK3CD. Biomed Pharmacother.

[53] Rahman MA, Salajegheh A, Smith RA (2015). MicroRNA-126 suppresses proliferation of undifferentiated (BRAF(V600E) and BRAF(WT)) thyroid carcinoma through targeting PIK3R2 gene and repressing PI3K-AKT proliferation-survival signalling pathway. Exp Cell Res.

[54] Boufraqech M, Zhang L, Jain M (2014). miR-145 suppresses thyroid cancer growth and metastasis and targets AKT3. Endocr Relat Cancer.

[55] Ramirez-Moya J, Wert-Lamas L, Santisteban P (2018). MicroRNA-146b promotes PI3K/AKT pathway hyperactivation and thyroid cancer progression by targeting PTEN. Oncogene.

[56] Xu Y, Han YF, Zhu SJ (2017). miRNA148a inhibits cell growth of papillary thyroid cancer through STAT3 and PI3K/AKT signaling pathways. Oncol Rep.

[57] Zhao J, Li Z, Chen Y (2019). MicroRNA766 inhibits papillary thyroid cancer progression by directly targeting insulin receptor substrate 2 and regulating the PI3K/Akt pathway. Int J Oncol.

[58] Min X, Liu K, Zhu H (2018). Long noncoding RNA LINC003121 inhibits proliferation and invasion of thyroid cancer cells by suppression of the phosphatidylinositol-3-kinase (PI3K)/Akt signaling pathway. Med Sci Monit.

[59] Wen J, Wang H, Dong T (2019). STAT3-induced upregulation of lncRNA ABHD11-AS1 promotes tumour progression in papillary thyroid carcinoma by regulating miR-1301-3p/STAT3 axis and PI3K/AKT signalling pathway. Cell Prolif.

[60] Li X, Li Q, Jin X (2019). Long non-coding RNA H19 knockdown inhibits the cell viability and promotes apoptosis of thyroid cancer cells through regulating the PI3K/AKT pathway. Exp Ther Med.

[61] Liu H, Deng H, Zhao Y (2018). LncRNA XIST/miR-34a axis modulates the cell proliferation and tumor growth of thyroid cancer through MET-PI3K-AKT signaling. J Exp Clin Cancer Res.

[62] Charles RP, Silva J, Iezza G (2014). Activating BRAF and PIK3CA mutations cooperate to promote anaplastic thyroid carcinogenesis. Mol Cancer Res.

[63] Gibson WJ, Ruan DT, Paulson VA (2017). Genomic heterogeneity and exceptional response to dual pathway inhibition in anaplastic thyroid cancer. Clin Cancer Res.

[64] Kotian S, Zhang L, Boufraqech M (2017). Dual inhibition of HDAC and tyrosine kinase signaling pathways with CUDC-907 inhibits thyroid cancer growth and metastases. Clin Cancer Res.

[65] Hoffman PC, Mauer AM, Vokes EE (2000). Lung cancer. Lancet.

[66] Perez-Ramirez C, Canadas-Garre M, Molina MA (2015). PTEN and PI3K/AKT in non-small-cell lung cancer. Pharmacogenomics.

[67] Kitagawa M, Liao PJ, Lee KH (2017). Dual blockade of the lipid kinase PIP4Ks and mitotic pathways leads to cancer-selective lethality. Nat Commun.

[68] Hu C, Zhou H, Liu Y (2019). ROCK1 promotes migration and invasion of nonsmallcell lung cancer cells through the PTEN/PI3K/FAK pathway. Int J Oncol.

[69] Chen B, Shen Z, Wu D (2019). Glutathione peroxidase 1 promotes NSCLC resistance to cisplatin via ROS-induced activation of PI3K/AKT pathway. Biomed Res Int.

[70] Li C, Lyu J, Meng QH (2017). MiR-93 promotes tumorigenesis and metastasis of non-small cell lung cancer cells by activating the PI3K/Akt pathway via inhibition of LKB1/PTEN/CDKN1A. J Cancer.

[71] Ma R, Zhu P, Liu S (2019). miR-496 suppress tumorigenesis via targeting BDNF-mediated PI3K/Akt signaling pathway in non-small cell lung cancer. Biochem Biophys Res Commun.

[72] Wu DM, Zhang T, Liu YB (2019). The PAX6-ZEB2 axis promotes metastasis and cisplatin resistance in non-small cell lung cancer through PI3K/AKT signaling. Cell Death Dis.

[73] Liu X, Lu X, Zhen F (2019). LINC00665 induces acquired resistance to gefitinib through recruiting EZH2 and activating PI3K/AKT pathway in NSCLC. Mol Ther Nucleic Acids.

[74] Li L, Li JC, Yang H (2018). Expansion of cancer stem cell pool initiates lung cancer recurrence before angiogenesis. Proc Natl Acad Sci USA.

[75] Saygin C, Matei D, Majeti R (2019). Targeting cancer stemness in the clinic: from hype to hope. Cell Stem Cell.

[76] Tripathi SC, Fahrmann JF, Celiktas M (2017). MCAM mediates chemoresistance in small-cell lung cancer via the PI3K/AKT/SOX2 signaling pathway. Cancer Res.

[77] Peng J, Wang Q, Liu H (2016). EPHA3 regulates the multidrug resistance of small cell lung cancer via the PI3K/BMX/STAT3 signaling pathway. Tumour Biol.

[78] Ngan HL, Wang L, Lo KW (2018). Genomic landscapes of EBV-associated nasopharyngeal carcinoma vs HPV-associated head and neck cancer. Cancers (Basel).

[79] Yu JH, Chen L, Yu JY (2019). PI3K-PKB-mTOR hyperactivation in relation to nasopharyngeal carcinoma progression and prognosis. J Cell Biochem.

[80] Zhao M, Luo R, Liu Y (2016). miR-3188 regulates nasopharyngeal carcinoma proliferation and chemosensitivity through a FOXO1-modulated positive feedback loop with mTOR-p-PI3K/AKT-c-JUN. Nat Commun.

[81] Chen J, Jiang C, Fu L (2019). CHL1 suppresses tumor growth and metastasis in nasopharyngeal carcinoma by repressing PI3K/AKT signaling pathway via interaction with Integrin beta1 and Merlin. Int J Biol Sci.

[82] Yu D, An X, Fan W (2019). PNUTS mediates ionizing radiation-induced CNE-2 nasopharyngeal carcinoma cell migration, invasion, and epithelial–mesenchymal transition via the PI3K/AKT signaling pathway. Onco Targets Ther.

[83] Liang Z, Liu Z, Cheng C (2019). VPS33B interacts with NESG1 to modulate EGFR/PI3K/AKT/c-Myc/P53/miR-133a-3p signaling and induce 5-fluorouracil sensitivity in nasopharyngeal carcinoma. Cell Death Dis.

[84] Ma R, Zhao LN, Yang H (2018). RNA binding motif protein 3 (RBM3) drives radioresistance in nasopharyngeal carcinoma by reducing apoptosis via the PI3K/AKT/Bcl-2 signaling pathway. Am J Transl Res.

[85] Hu Q, Lin X, Ding L (2018). ARHGAP42 promotes cell migration and invasion involving PI3K/Akt signaling pathway in nasopharyngeal carcinoma. Cancer Med.

[86] Wang X, Jin Q, Wang X (2019). LncRNA ZFAS1 promotes proliferation and migration and inhibits apoptosis in nasopharyngeal carcinoma via the PI3K/AKT pathway in vitro. Cancer Biomark.

[87] Zhang P, Lu X, Shi Z (2019). miR-205-5p regulates epithelial–mesenchymal transition by targeting PTEN via PI3K/AKT signaling pathway in cisplatin-resistant nasopharyngeal carcinoma cells. Gene.

[88] Chu EA, Kim YJ (2008). Laryngeal cancer: diagnosis and preoperative work-up. Otolaryngol Clin North Am.

[89] Mehanna H, Beech T, Nicholson T (2013). Prevalence of human papillomavirus in oropharyngeal and nonoropharyngeal head and neck cancer-systematic review and meta-analysis of trends by time and region. Head Neck.

[90] Lui VW, Hedberg ML, Li H (2013). Frequent mutation of the PI3K pathway in head and neck cancer defines predictive biomarkers. Cancer Discov.

[91] Stransky N, Egloff AM, Tward AD (2011). The mutational landscape of head and neck squamous cell carcinoma. Science.

[92] Agrawal N, Frederick MJ, Pickering CR (2011). Exome sequencing of head and neck squamous cell carcinoma reveals inactivating mutations in NOTCH1. Science.

[93] Cancer Genome Atlas Network (2015). Comprehensive genomic characterization of head and neck squamous cell carcinomas. Nature.

[94] Zhu Y, Yan L, Zhu W (2019). MMP2/3 promote the growth and migration of laryngeal squamous cell carcinoma via PI3K/Akt-NF-kappaB-mediated epithelial–mesenchymal transformation. J Cell Physiol.

[95] Tian L, Tao ZZ, Ye HP (2018). Over-expression of MEOX2 promotes apoptosis through inhibiting the PI3K/Akt pathway in laryngeal cancer cells. Neoplasma.

[96] Ye D, Zhou C, Deng H (2019). MicroRNA-145 inhibits growth of laryngeal squamous cell carcinoma by targeting the PI3K/Akt signaling pathway. Cancer Manag Res.

[97] Si F, Sun J, Wang C (2017). MicroRNA-138 suppresses cell proliferation in laryngeal squamous cell carcinoma via inhibiting EZH2 and PI3K/AKT signaling. Exp Ther Med.

[98] Lin DC, Hao JJ, Nagata Y (2014). Genomic and molecular characterization of esophageal squamous cell carcinoma. Nat Genet.

[99] Munari FF, Cruvinel-Carloni A, Lacerda CF (2018). PIK3CA mutations are frequent in esophageal squamous cell carcinoma associated with chagasic megaesophagus and are associated with a worse patient outcome. Infect Agent Cancer.

[100] Wang H, Yang X, Guo Y (2019). HERG1 promotes esophageal squamous cell carcinoma growth and metastasis through TXNDC5 by activating the PI3K/AKT pathway. J Exp Clin Cancer Res.

[101] Hou G, Zhao Q, Zhang M (2019). LSD1 regulates Notch and PI3K/Akt/mTOR pathways through binding the promoter regions of Notch target genes in esophageal squamous cell carcinoma. Onco Targets Ther.

[102] Jia Y, Xiao Z, Gongsun X (2018). CEP55 promotes the proliferation, migration and invasion of esophageal squamous cell carcinoma via the PI3K/Akt pathway. Onco Targets Ther.

[103] Nie C, Qin X, Li X (2019). CACNA2D3 enhances the chemosensitivity of esophageal squamous cell carcinoma to cisplatin via inducing Ca(2+)-mediated apoptosis and suppressing PI3K/Akt pathways. Front Oncol.

[104] He Y, Mingyan E, Wang C (2019). CircVRK1 regulates tumor progression and radioresistance in esophageal squamous cell carcinoma by regulating miR-624-3p/PTEN/PI3K/AKT signaling pathway. Int J Biol Macromol.

[105] Wang G, Sun J, Zhao H (2018). Long non-coding RNA (lncRNA) growth arrest specific 5 (GAS5) suppresses esophageal squamous cell carcinoma cell proliferation and migration by inactivating phosphatidylinositol 3-kinase (PI3K)/AKT/mammalian target of rapamycin (mTOR) signaling pathway. Med Sci Monit.

[106] Li WQ, Zhang JY, Ma JL (2019). Effects of Helicobacter pylori treatment and vitamin and garlic supplementation on gastric cancer incidence and mortality: follow-up of a randomized intervention trial. BMJ.

[107] Fang WL, Huang KH, Lan YT (2016). Mutations in PI3K/AKT pathway genes and amplifications of PIK3CA are associated with patterns of recurrence in gastric cancers. Oncotarget.

[108] Ito C, Nishizuka SS, Ishida K (2017). Analysis of PIK3CA mutations and PI3K pathway proteins in advanced gastric cancer. J Surg Res.

[109] Liu JY, Jiang L, He T (2019). NETO2 promotes invasion and metastasis of gastric cancer cells via activation of PI3K/Akt/NF-kappaB/Snail axis and predicts outcome of the patients. Cell Death Dis.

[110] Lin JX, Xie XS, Weng XF (2019). UFM1 suppresses invasive activities of gastric cancer cells by attenuating the expres7sion of PDK1 through PI3K/AKT signaling. J Exp Clin Cancer Res.

[111] Wang J, Zhang Y, Dou Z (2019). Knockdown of STIL suppresses the progression of gastric cancer by down-regulating the IGF-1/PI3K/AKT pathway. J Cell Mol Med.

[112] Li Q, Ge Y, Chen X (2019). LEM domain containing 1 promotes proliferation via activating the PI3K/Akt signaling pathway in gastric cancer. J Cell Biochem.

[113] Song SZ, Lin S, Liu JN (2019). Targeting of SPP1 by microRNA-340 inhibits gastric cancer cell epithelial–mesenchymal transition through inhibition of the PI3K/AKT signaling pathway. J Cell Physiol.

[114] Xiong J, Li Z, Zhang Y (2016). PRL-3 promotes the peritoneal metastasis of gastric cancer through the PI3K/Akt signaling pathway by regulating PTEN. Oncol Rep.

[115] Lu W, Xu Z, Zhang M (2014). MiR-19a promotes epithelial–mesenchymal transition through PI3K/AKT pathway in gastric cancer. Int J Clin Exp Pathol.

[116] Wang P, Guan Q, Zhou D (2018). miR-21 inhibitors modulate biological functions of gastric cancer cells via PTEN/PI3K/mTOR pathway. DNA Cell Biol.

[117] Cao W, Yang W, Fan R (2014). miR-34a regulates cisplatin-induce gastric cancer cell death by modulating PI3K/AKT/survivin pathway. Tumour Biol.

[118] Cheng Y, Li Y, Liu D (2014). miR-137 effects on gastric carcinogenesis are mediated by targeting Cox-2-activated PI3K/AKT signaling pathway. FEBS Lett.

[119] Li NA, Wang W, Xu B (2016). miR-196b regulates gastric cancer cell proliferation and invasion via PI3K/AKT/mTOR signaling pathway. Oncol Lett.

[120] Zhu K, Ren Q, Zhao Y (2019). lncRNA MALAT1 overexpression promotes proliferation, migration and invasion of gastric cancer by activating the PI3K/AKT pathway. Oncol Lett.

[121] Cen D, Huang H, Yang L (2019). Long noncoding RNA STXBP5-AS1 inhibits cell proliferation, migration, and invasion through inhibiting the PI3K/AKT signaling pathway in gastric cancer cells. Onco Targets Ther.

[122] Li JF, Li WH, Xue LL (2019). Long non-coding RNA PICART1 inhibits cell proliferation by regulating the PI3K/AKT and MAPK/ERK signaling pathways in gastric cancer. Eur Rev Med Pharmacol Sci.

[123] Wieszczy P, Kaminski MF, Franczyk R (2019). Colorectal cancer incidence and mortality after removal of adenomas during screening colonoscopies. Gastroenterology pii.

[124] Jin J, Shi Y, Zhang S (2020). PIK3CA mutation and clinicopathological features of colorectal cancer: a systematic review and meta-analysis. Acta Oncol.

[125] Ma J, Sun X, Wang Y (2019). Fibroblast-derived CXCL12 regulates PTEN expression and is associated with the proliferation and invasion of colon cancer cells via PI3k/Akt signaling. Cell Commun Signal.

[126] Karki R, Man SM, Malireddi RKS (2016). NLRC3 is an inhibitory sensor of PI3K-mTOR pathways in cancer. Nature.

[127] Qi J, Yu Y, Akilli Ozturk O (2016). New Wnt/beta-catenin target genes promote experimental metastasis and migration of colorectal cancer cells through different signals. Gut.

[128] Valeri N, Braconi C, Gasparini P (2014). MicroRNA-135b promotes cancer progression by acting as a downstream effector of oncogenic pathways in colon cancer. Cancer Cell.

[129] Tsai KW, Lo YH, Liu H (2018). Linc00659, a long noncoding RNA, acts as novel oncogene in regulating cancer cell growth in colorectal cancer. Mol Cancer.

[130] Ellis BC, Graham LD (1843). Molloy PL (2014) CRNDE, a long non-coding RNA responsive to insulin/IGF signaling, regulates genes involved in central metabolism. Biochim Biophys Acta.

[131] Goodwin R, Jonker D, Chen E (2019). A phase Ib study of a PI3Kinase inhibitor BKM120 in combination with panitumumab in patients with KRAS wild-type advanced colorectal cancer. Invest New Drugs.

[132] Hirota S, Isozaki K, Moriyama Y (1998). Gain-of-function mutations of c-kit in human gastrointestinal stromal tumors. Science.

[133] Joensuu H, Rutkowski P, Nishida T (2015). KIT and PDGFRA mutations and the risk of GI stromal tumor recurrence. J Clin Oncol.

[134] Ohshima K, Fujiya K, Nagashima T (2019). Driver gene alterations and activated signaling pathways toward malignant progression of gastrointestinal stromal tumors. Cancer Sci.

[135] Rossi S, Ou W, Tang D (2006). Gastrointestinal stromal tumours overexpress fatty acid synthase. J Pathol.

[136] Li CF, Fang FM, Chen YY (2017). Overexpressed fatty acid synthase in gastrointestinal stromal tumors: targeting a progression-associated metabolic driver enhances the antitumor effect of imatinib. Clin Cancer Res.

[137] Long ZW, Wu JH, Cai H (2018). MiR-374b promotes proliferation and inhibits apoptosis of human GIST cells by inhibiting PTEN through activation of the PI3K/Akt pathway. Mol Cells.

[138] Zook P, Pathak HB, Belinsky MG (2017). Combination of imatinib mesylate and AKT inhibitor provides synergistic effects in preclinical study of gastrointestinal stromal tumor. Clin Cancer Res.

[139] He X, Zhu Z, Johnson C (2008). PIK3IP1, a negative regulator of PI3K, suppresses the development of hepatocellular carcinoma. Cancer Res.

[140] Chen H, Wong CC, Liu D (2019). APLN promotes hepatocellular carcinoma through activating PI3K/Akt pathway and is a druggable target. Theranostics.

[141] Fang Y, Xue JL, Shen Q (2012). MicroRNA-7 inhibits tumor growth and metastasis by targeting the phosphoinositide 3-kinase/Akt pathway in hepatocellular carcinoma. Hepatology.

[142] Wang Z, Luo Y (2019). MicroRNA-367 promotes progression of hepatocellular carcinoma through PTEN /PI3K/AKT signaling pathway. Biosci Rep..

[143] Xu Q, Liu X, Liu Z (2017). MicroRNA-1296 inhibits metastasis and epithelial–mesenchymal transition of hepatocellular carcinoma by targeting SRPK1-mediated PI3K/AKT pathway. Mol Cancer.

[144] Du W, Zhang X, Wan Z (2019). miR-3691-5p promotes hepatocellular carcinoma cell migration and invasion through activating PI3K/Akt signaling by targeting PTEN. Onco Targets Ther.

[145] Huang JL, Cao SW, Ou QS (2018). The long non-coding RNA PTTG3P promotes cell growth and metastasis via up-regulating PTTG1 and activating PI3K/AKT signaling in hepatocellular carcinoma. Mol Cancer.

[146] Zheng YF, Zhang XY, Bu YZ (2019). LINC01133 aggravates the progression of hepatocellular carcinoma by activating the PI3K/AKT pathway. J Cell Biochem.

[147] Zhao S, Cao Y, Liu SB (2016). The E545K mutation of PIK3CA promotes gallbladder carcinoma progression through enhanced binding to EGFR. J Exp Clin Cancer Res.

[148] Li M, Liu F, Zhang F (2019). Genomic ERBB2/ERBB3 mutations promote PD-L1-mediated immune escape in gallbladder cancer: a whole-exome sequencing analysis. Gut.

[149] Zhang F, Xiang S, Cao Y (2017). EIF3D promotes gallbladder cancer development by stabilizing GRK2 kinase and activating PI3K-AKT signaling pathway. Cell Death Dis.

[150] Zhang Z, Zheng X, Li J (2019). Overexpression of UBR5 promotes tumor growth in gallbladder cancer via PTEN/PI3K/Akt signal pathway. J Cell Biochem.

[151] Hao J, Yang Z, Wang L (2017). Downregulation of BRD4 inhibits gallbladder cancer proliferation and metastasis and induces apoptosis via PI3K/AKT pathway. Int J Oncol.

[152] Li H, Zhang Y, Hai J (2018). Knockdown of TRIM31 suppresses proliferation and invasion of gallbladder cancer cells by down-regulating MMP2/9 through the PI3K/Akt signaling pathway. Biomed Pharmacother.

[153] Cai Q, Wang ZQ, Wang SH (2016). Upregulation of long non-coding RNA LINC00152 by SP1 contributes to gallbladder cancer cell growth and tumor metastasis via PI3K/AKT pathway. Am J Transl Res.

[154] Shin EJ, Canto MI (2012). Pancreatic cancer screening. Gastroenterol Clin N Am.

[155] Delpu Y, Hanoun N, Lulka H (2011). Genetic and epigenetic alterations in pancreatic carcinogenesis. Curr Genomics.

[156] Carter H, Samayoa J, Hruban RH (2010). Prioritization of driver mutations in pancreatic cancer using cancer-specific high-throughput annotation of somatic mutations (CHASM). Cancer Biol Ther.

[157] di Magliano MP, Logsdon CD (2013). Roles for KRAS in pancreatic tumor development and progression. Gastroenterology.

[158] Bournet B, Buscail C, Muscari F (2016). Targeting KRAS for diagnosis, prognosis, and treatment of pancreatic cancer: hopes and realities. Eur J Cancer.

[159] Bittoni A, Mandolesi A, Andrikou K (2015). HER family receptor expression and prognosis in pancreatic cancer. Int J Biol Markers.

[160] Baer R, Cintas C, Dufresne M (2014). Pancreatic cell plasticity and cancer initiation induced by oncogenic Kras is completely dependent on wild-type PI 3-kinase p110alpha. Genes Dev.

[161] King H, Thillai K, Whale A (2017). PAK4 interacts with p85 alpha: implications for pancreatic cancer cell migration. Sci Rep.

[162] Yan X, Hui Y, Hua Y (2019). EG-VEGF silencing inhibits cell proliferation and promotes cell apoptosis in pancreatic carcinoma via PI3K/AKT/mTOR signaling pathway. Biomed Pharmacother.

[163] Fu Y, Yao N, Ding D (2020). TMEM158 promotes pancreatic cancer aggressiveness by activation of TGFbeta1 and PI3K/AKT signaling pathway. J Cell Physiol.

[164] Xiong J, Wang D, Wei A (2017). Deregulated expression of miR-107 inhibits metastasis of PDAC through inhibition PI3K/Akt signaling via caveolin-1 and PTEN. Exp Cell Res.

[165] Qiao X, Lv SX, Qiao Y (2018). Long noncoding RNA ABHD11-AS1 predicts the prognosis of pancreatic cancer patients and serves as a promoter by activating the PI3K-AKT pathway. Eur Rev Med Pharmacol Sci.

[166] Zhang Y, Zhang R, Luo G (2018). Long noncoding RNA SNHG1 promotes cell proliferation through PI3K/AKT signaling pathway in pancreatic ductal adenocarcinoma. J Cancer.

[167] Wang L, Wang F, Na L (2018). LncRNA AB209630 inhibits gemcitabine resistance cell proliferation by regulating PI3K/AKT signaling in pancreatic ductal adenocarcinoma. Cancer Biomark.

[168] Croessmann S, Formisano L, Kinch LN (2019). Combined blockade of activating ERBB2 mutations and ER results in synthetic lethality of ER+/HER2 mutant breast cancer. Clin Cancer Res.

[169] Nixon MJ, Formisano L, Mayer IA (2019). PIK3CA and MAP3K1 alterations imply luminal A status and are associated with clinical benefit from pan-PI3K inhibitor buparlisib and letrozole in ER+ metastatic breast cancer. NPJ Breast Cancer.

[170] Adamo B, Deal AM, Burrows E (2011). Phosphatidylinositol 3-kinase pathway activation in breast cancer brain metastases. Breast Cancer Res.

[171] Ippen FM, Alvarez-Breckenridge CA, Kuter BM (2019). The dual PI3K/mTOR pathway inhibitor GDC-0084 achieves antitumor activity in PIK3CA-mutant breast cancer brain metastases. Clin Cancer Res.

[172] Goffin V (2017). Prolactin receptor targeting in breast and prostate cancers: new insights into an old challenge. Pharmacol Ther.

[173] Ikink GJ, Boer M, Bakker ER (2016). IRS4 induces mammary tumorigenesis and confers resistance to HER2-targeted therapy through constitutive PI3K/AKT-pathway hyperactivation. Nat Commun.

[174] Choi HJ, Jin S, Cho H (2019). CDK12 drives breast tumor initiation and trastuzumab resistance via WNT and IRS1-ErbB-PI3K signaling. EMBO Rep.

[175] Zhou J, Pei Y, Chen G (2018). SPC24 Regulates breast cancer progression by PI3K/AKT signaling. Gene.

[176] Zhang S, Chung WC, Wu G (2015). Manic fringe promotes a claudin-low breast cancer phenotype through notch-mediated PIK3CG induction. Cancer Res.

[177] Liu L, Meng T, Zheng X (2019). Transgelin 2 promotes paclitaxel resistance, migration, and invasion of breast cancer by directly interacting with PTEN and activating PI3K/Akt/GSK-3beta pathway. Mol Cancer Ther.

[178] Nan H, Han L, Ma J (2018). STX3 represses the stability of the tumor suppressor PTEN to activate the PI3K-Akt-mTOR signaling and promotes the growth of breast cancer cells. Biochim Biophys Acta Mol Basis Dis.

[179] Mehta GA, Parker JS, Silva GO (2017). Amplification of SOX4 promotes PI3K/Akt signaling in human breast cancer. Breast Cancer Res Treat.

[180] He LF, Xu HW, Chen M (2017). Activated-PAK4 predicts worse prognosis in breast cancer and promotes tumorigenesis through activation of PI3K/AKT signaling. Oncotarget.

[181] Chen M, Zhang H, Zhang G (2018). Targeting TPX2 suppresses proliferation and promotes apoptosis via repression of the PI3k/AKT/P21 signaling pathway and activation of p53 pathway in breast cancer. Biochem Biophys Res Commun.

[182] Zhu M, Wang X, Gu Y (2019). MEG3 overexpression inhibits the tumorigenesis of breast cancer by downregulating miR-21 through the PI3K/Akt pathway. Arch Biochem Biophys.

[183] Li N, Miao Y, Shan Y (2017). MiR-106b and miR-93 regulate cell progression by suppression of PTEN via PI3K/Akt pathway in breast cancer. Cell Death Dis.

[184] Miao Y, Zheng W, Li N (2017). MicroRNA-130b targets PTEN to mediate drug resistance and proliferation of breast cancer cells via the PI3K/Akt signaling pathway. Sci Rep.

[185] Wang F, Li L, Chen Z (2016). MicroRNA-214 acts as a potential oncogene in breast cancer by targeting the PTEN-PI3K/Akt signaling pathway. Int J Mol Med.

[186] Han J, Yu J, Dai Y (2019). Overexpression of miR-361-5p in triple-negative breast cancer (TNBC) inhibits migration and invasion by targeting RQCD1 and inhibiting the EGFR/PI3K/Akt pathway. Bosn J Basic Med Sci.

[187] Chen X, Wang YW, Xing AY (2016). Suppression of SPIN1-mediated PI3K-Akt pathway by miR-489 increases chemosensitivity in breast cancer. J Pathol.

[188] Zhao Y, Pang W, Yang N (2018). MicroRNA-511 inhibits malignant behaviors of breast cancer by directly targeting SOX9 and regulating the PI3K/Akt pathway. Int J Oncol.

[189] Mutlu M, Saatci O, Ansari SA (2016). miR-564 acts as a dual inhibitor of PI3K and MAPK signaling networks and inhibits proliferation and invasion in breast cancer. Sci Rep.

[190] Li Z, Qian J, Li J (2019). Knockdown of lncRNA-HOTAIR downregulates the drug-resistance of breast cancer cells to doxorubicin via the PI3K/AKT/mTOR signaling pathway. Exp Ther Med.

[191] Xu S, Sui S, Zhang J (2015). Downregulation of long noncoding RNA MALAT1 induces epithelial-to-mesenchymal transition via the PI3K-AKT pathway in breast cancer. Int J Clin Exp Pathol.

[192] Kuo KT, Mao TL, Jones S (2009). Frequent activating mutations of PIK3CA in ovarian clear cell carcinoma. Am J Pathol.

[193] Verhaak RG, Tamayo P, Yang JY (2013). Prognostically relevant gene signatures of high-grade serous ovarian carcinoma. J Clin Invest.

[194] Wei X, Jia Y, Lou H (2019). Targeting YAP suppresses ovarian cancer progression through regulation of the PI3K/Akt/mTOR pathway. Oncol Rep.

[195] Wu Y, Cai Q, Li W (2019). Active PKG II inhibited the growth and migration of ovarian cancer cells through blocking Raf/MEK and PI3K/Akt signaling pathways. Biosci Rep.

[196] Gao T, Zhang X, Zhao J (2020). SIK2 promotes reprogramming of glucose metabolism through PI3K/AKT/HIF-1alpha pathway and Drp1-mediated mitochondrial fission in ovarian cancer. Cancer Lett.

[197] Guo Q, Zhu L, Wang C (2019). SERPIND1 affects the malignant biological behavior of epithelial ovarian cancer via the PI3K/AKT pathway: a mechanistic study. Front Oncol.

[198] Li GC, Qin XL, Song HH (2019). Upregulated microRNA-15b alleviates ovarian cancer through inhitbition of the PI3K/Akt pathway by targeting LPAR3. J Cell Physiol.

[199] Liu HY, Zhang YY, Zhu BL (2019). miR-21 regulates the proliferation and apoptosis of ovarian cancer cells through PTEN/PI3K/AKT. Eur Rev Med Pharmacol Sci.

[200] Wuerkenbieke D, Wang J, Li Y (2015). miRNA-150 downregulation promotes pertuzumab resistance in ovarian cancer cells via AKT activation. Arch Gynecol Obstet.

[201] Fu X, Li Y, Alvero A (2016). MicroRNA-222-3p/GNAI2/AKT axis inhibits epithelial ovarian cancer cell growth and associates with good overall survival. Oncotarget.

[202] Zhang Z, Zhang L, Wang B (2020). MiR-337-3p suppresses proliferation of epithelial ovarian cancer by targeting PIK3CA and PIK3CB. Cancer Lett.

[203] Wang W, Ren F, Wu Q (2014). MicroRNA-497 suppresses angiogenesis by targeting vascular endothelial growth factor A through the PI3K/AKT and MAPK/ERK pathways in ovarian cancer. Oncol Rep.

[204] Wu D, Lu P, Mi X (2018). Downregulation of miR-503 contributes to the development of drug resistance in ovarian cancer by targeting PI3K p85. Arch Gynecol Obstet.

[205] Li C, Yu S, Wu S (2019). MicroRNA-936 targets FGF2 to inhibit epithelial ovarian cancer aggressiveness by deactivating the PI3K/Akt pathway. Onco Targets Ther.

[206] Jin Y, Feng SJ, Qiu S (2017). LncRNA MALAT1 promotes proliferation and metastasis in epithelial ovarian cancer via the PI3K-AKT pathway. Eur Rev Med Pharmacol Sci.

[207] Li J, Feng L, Tian C (2018). Long noncoding RNA-JPX predicts the poor prognosis of ovarian cancer patients and promotes tumor cell proliferation, invasion and migration by the PI3K/Akt/mTOR signaling pathway. Eur Rev Med Pharmacol Sci.

[208] Kurman RJ, Shih Ie M (2010). The origin and pathogenesis of epithelial ovarian cancer: a proposed unifying theory. Am J Surg Pathol.

[209] Gungorduk K, Ertas IE, Ozdemir A (2015). Prognostic significance of retroperitoneal lymphadenectomy, preoperative neutrophil lymphocyte ratio and platelet lymphocyte ratio in primary fallopian tube carcinoma: a multicenter study. Cancer Res Treat.

[210] Nakamura K, Nakayama K, Ishikawa N (2018). Reconstitution of high-grade serous ovarian carcinoma from primary fallopian tube secretory epithelial cells. Oncotarget.

[211] Snijders AM, Nowee ME, Fridlyand J (2003). Genome-wide-array-based comparative genomic hybridization reveals genetic homogeneity and frequent copy number increases encompassing CCNE1 in fallopian tube carcinoma. Oncogene.

[212] Jiang W, He T, Liu S (2018). The PIK3CA E542K and E545K mutations promote glycolysis and proliferation via induction of the beta-catenin/SIRT3 signaling pathway in cervical cancer. J Hematol Oncol.

[213] Qin Y, Tang X, Liu M (2016). Tumor-suppressor gene nbpf1 inhibits invasion and PI3K/mTOR signaling in cervical cancer cells. Oncol Res.

[214] Guo Q, Xiong Y, Song Y (2019). ARHGAP17 suppresses tumor progression and up-regulates P21 and P27 expression via inhibiting PI3K/AKT signaling pathway in cervical cancer. Gene.

[215] Li YJ, Wang Y, Wang YY (2019). MicroRNA-99b suppresses human cervical cancer cell activity by inhibiting the PI3K/AKT/mTOR signaling pathway. J Cell Physiol.

[216] Mei Q, Li X, Zhang K (2017). Genetic and methylation-induced loss of miR-181a2/181b2 within chr9q33.3 facilitates tumor growth of cervical cancer through the PIK3R3/Akt/FoxO signaling pathway. Clin Cancer Res.

[217] Lu R, Yang Z, Xu G (2018). miR-338 modulates proliferation and autophagy by PI3K/AKT/mTOR signaling pathway in cervical cancer. Biomed Pharmacother.

[218] Teng P, Jiao Y, Hao M (2018). microRNA-383 suppresses the PI3K-AKT-MTOR signaling pathway to inhibit development of cervical cancer via down-regulating PARP2. J Cell Biochem.

[219] Xu J, Zhu W, Chen L (2018). MicroRNA433 inhibits cell growth and induces apoptosis in human cervical cancer through PI3K/AKT signaling by targeting FAK. Oncol Rep.

[220] Juan C, Hua Q, Ruping Z (2018). miRNA-489 as a biomarker in diagnosis and treatment of cervical cancer. Bratisl Lek Listy.

[221] Zhang D, Sun G, Zhang H (2017). Long non-coding RNA ANRIL indicates a poor prognosis of cervical cancer and promotes carcinogenesis via PI3K/Akt pathways. Biomed Pharmacother.

[222] Yang HY, Huang CP, Cao MM (2018). Long non-coding RNA CRNDE may be associated with poor prognosis by promoting proliferation and inhibiting apoptosis of cervical cancer cells through targeting PI3K/AKT. Neoplasma.

[223] Guo HM, Yang SH, Zhao SZ (2018). LncRNA NEAT1 regulates cervical carcinoma proliferation and invasion by targeting AKT/PI3K. Eur Rev Med Pharmacol Sci.

[224] Yan SP, Chu DX, Qiu HF (2019). LncRNA LINC01305 silencing inhibits cell epithelial–mesenchymal transition in cervical cancer by inhibiting TNXB-mediated PI3K/Akt signalling pathway. J Cell Mol Med.

[225] Lee CM, Fuhrman CB, Planelles V (2006). Phosphatidylinositol 3-kinase inhibition by LY294002 radiosensitizes human cervical cancer cell lines. Clin Cancer Res.

[226] Liu Y, Cui B, Qiao Y (2011). Phosphoinositide-3-kinase inhibition enhances radiosensitization of cervical cancer in vivo. Int J Gynecol Cancer.

[227] Malentacchi F, Turrini I, Sorbi F (2019). Pilot investigation of the mutation profile of PIK3CA/PTEN genes (PI3K pathway) in grade 3 endometrial cancer. Oncol Rep.

[228] Qiu H, Li J, Clark LH (2016). JQ1 suppresses tumor growth via PTEN/PI3K/AKT pathway in endometrial cancer. Oncotarget.

[229] Zhang Y, Goodfellow R, Li Y (2015). NEDD4 ubiquitin ligase is a putative oncogene in endometrial cancer that activates IGF-1R/PI3K/Akt signaling. Gynecol Oncol.

[230] Wang X, Li Y, Wan L (2019). Downregulation of PDCD4 induced by progesterone is mediated by the PI3K/AKT signaling pathway in human endometrial cancer cells. Oncol Rep.

[231] Zhang S, Wang M, Li Q (2017). MiR-101 reduces cell proliferation and invasion and enhances apoptosis in endometrial cancer via regulating PI3K/Akt/mTOR. Cancer Biomark.

[232] Zhu L, Wang X, Wang T (2019). miR4943p promotes the progression of endometrial cancer by regulating the PTEN/PI3K/AKT pathway. Mol Med Rep.

[233] Zhang XH, Li M, Kang YJ (2019). Long non-coding RNA LINP1 functions as an oncogene in endometrial cancer progression by regulating the PI3K/AKT signaling pathway. Eur Rev Med Pharmacol Sci.

[234] Sun KX, Wu DD, Chen S (2017). LncRNA MEG3 inhibit endometrial carcinoma tumorigenesis and progression through PI3K pathway. Apoptosis.

[235] Barry MJ, Simmons LH (2017). Prevention of prostate cancer morbidity and mortality: primary prevention and early detection. Med Clin N Am.

[236] Wise HM, Hermida MA, Leslie NR (2017). Prostate cancer, PI3K, PTEN and prognosis. Clin Sci (Lond).

[237] Zhu W, Shao Y, Yang M (2016). Asparaginyl endopeptidase promotes proliferation and invasiveness of prostate cancer cells via PI3K/AKT signaling pathway. Gene.

[238] Wu X, Xiao Y, Yan W (2019). The human oncogene SCL/TAL1 interrupting locus (STIL) promotes tumor growth through MAPK/ERK, PI3K/Akt and AMPK pathways in prostate cancer. Gene.

[239] Quan Y, Wang N, Chen Q (2015). SIRT3 inhibits prostate cancer by destabilizing oncoprotein c-MYC through regulation of the PI3K/Akt pathway. Oncotarget.

[240] Henderson V, Smith B, Burton LJ (2015). Snail promotes cell migration through PI3K/AKT-dependent Rac1 activation as well as PI3K/AKT-independent pathways during prostate cancer progression. Cell Adh Migr.

[241] Offermann A, Vlasic I, Syring I (2017). MED15 overexpression in prostate cancer arises during androgen deprivation therapy via PI3K/mTOR signaling. Oncotarget.

[242] Zhou Y, Gu P, Li J (2017). Suppression of STIM1 inhibits the migration and invasion of human prostate cancer cells and is associated with PI3K/Akt signaling inactivation. Oncol Rep.

[243] Wei A, Fan B, Zhao Y (2016). ST6Gal-I overexpression facilitates prostate cancer progression via the PI3K/Akt/GSK-3beta/beta-catenin signaling pathway. Oncotarget.

[244] Talesa VN, Ferri I, Bellezza G (2017). Glyoxalase 2 Is involved in human prostate cancer progression as part of a mechanism driven By PTEN/PI3K/AKT/mTOR signaling with involvement of PKM2 and ERalpha. Prostate.

[245] Han G, Zhang X, Liu P (2018). Knockdown of anti-silencing function 1B histone chaperone induces cell apoptosis via repressing PI3K/Akt pathway in prostate cancer. Int J Oncol.

[246] Liang F, Yue J, Wang J (2015). GPCR48/LGR4 promotes tumorigenesis of prostate cancer via PI3K/Akt signaling pathway. Med Oncol.

[247] Chen C, Cai Q, He W (2017). AP4 modulated by the PI3K/AKT pathway promotes prostate cancer proliferation and metastasis of prostate cancer via upregulating L-plastin. Cell Death Dis.

[248] Shao G, Liu Y, Ma T (2018). GCN5 inhibition prevents IL-6-induced prostate cancer metastases through PI3K/PTEN/Akt signaling by inactivating Egr-1. Biosci Rep.

[249] Tan M, Xu J, Siddiqui J (2016). Depletion of SAG/RBX2 E3 ubiquitin ligase suppresses prostate tumorigenesis via inactivation of the PI3K/AKT/mTOR axis. Mol Cancer.

[250] Chang YL, Zhou PJ, Wei L (2015). MicroRNA-7 inhibits the stemness of prostate cancer stem-like cells and tumorigenesis by repressing KLF4/PI3K/Akt/p21 pathway. Oncotarget.

[251] Yang J, Song Q, Cai Y (2015). RLIP76-dependent suppression of PI3K/AKT/Bcl-2 pathway by miR-101 induces apoptosis in prostate cancer. Biochem Biophys Res Commun.

[252] Xu S, Ge J, Zhang Z (2017). MiR-129 inhibits cell proliferation and metastasis by targeting ETS1 via PI3K/AKT/mTOR pathway in prostate cancer. Biomed Pharmacother.

[253] Tang Y, Pan J, Huang S (2018). Downregulation of miR-133a-3p promotes prostate cancer bone metastasis via activating PI3K/AKT signaling. J Exp Clin Cancer Res.

[254] Wang Y, Shao N, Mao X (2016). MiR-4638-5p inhibits castration resistance of prostate cancer through repressing Kidins220 expression and PI3K/AKT pathway activity. Oncotarget.

[255] Wang YC, He WY, Dong CH (2019). lncRNA HCG11 regulates cell progression by targeting miR-543 and regulating AKT/mTOR pathway in prostate cancer. Cell Biol Int.

[256] Xu S, Yi XM, Tang CP (2016). Long non-coding RNA ATB promotes growth and epithelial–mesenchymal transition and predicts poor prognosis in human prostate carcinoma. Oncol Rep.

[257] Shen G, Jiang M, Pu J (2018). Dual inhibition of BRD4 and PI3K by SF2523 suppresses human prostate cancer cell growth in vitro and in vivo. Biochem Biophys Res Commun.

[258] Damayanti NP, Budka JA, Khella HWZ (2018). Therapeutic targeting of TFE3/IRS-1/PI3K/mTOR axis in translocation renal cell carcinoma. Clin Cancer Res.

[259] Lin Y, Yang Z, Xu A (2015). PIK3R1 negatively regulates the epithelial–mesenchymal transition and stem-like phenotype of renal cancer cells through the AKT/GSK3beta/CTNNB1 signaling pathway. Sci Rep.

[260] Lin A, Piao HL, Zhuang L (2014). FoxO transcription factors promote AKT Ser473 phosphorylation and renal tumor growth in response to pharmacologic inhibition of the PI3K-AKT pathway. Cancer Res.

[261] Huang B, Fu SJ, Fan WZ (2016). PKCepsilon inhibits isolation and stemness of side population cells via the suppression of ABCB1 transporter and PI3K/Akt, MAPK/ERK signaling in renal cell carcinoma cell line 769P. Cancer Lett.

[262] Zhao Z, Liu H, Hou J (2017). Tumor protein D52 (TPD52) inhibits growth and metastasis in renal cell carcinoma cells through the PI3K/Akt signaling pathway. Oncol Res.

[263] Liu S, Ma X, Ai Q (2013). NOTCH1 functions as an oncogene by regulating the PTEN/PI3K/AKT pathway in clear cell renal cell carcinoma. Urol Oncol.

[264] Zhang GW, Tian X, Li Y (2018). Down-regulation of ETS2 inhibits the invasion and metastasis of renal cell carcinoma cells by inducing EMT via the PI3K/Akt signaling pathway. Biomed Pharmacother.

[265] Liu GL, Yang HJ, Liu B (2017). Effects of microRNA-19b on the proliferation, apoptosis, and migration of wilms' tumor cells via the PTEN/PI3K/AKT signaling pathway. J Cell Biochem.

[266] Lian JH, Wang WH, Wang JQ (2013). MicroRNA-122 promotes proliferation, invasion and migration of renal cell carcinoma cells through the PI3K/Akt signaling pathway. Asian Pac J Cancer Prev.

[267] Fu JH, Yang S, Nan CJ (2018). MiR-182 affects renal cancer cell proliferation, apoptosis, and invasion by regulating PI3K/AKT/mTOR signaling pathway. Eur Rev Med Pharmacol Sci.

[268] Pan Y, Hu J, Ma J (2018). MiR-193a-3p and miR-224 mediate renal cell carcinoma progression by targeting alpha-2,3-sialyltransferase IV and the phosphatidylinositol 3 kinase/Akt pathway. Mol Carcinog.

[269] Sun P, Wang L, Lu Y (2016). MicroRNA-195 targets VEGFR2 and has a tumor suppressive role in ACHN cells via PI3K/Akt and Raf/MEK/ERK signaling pathways. Int J Oncol.

[270] Li Z, Ma Z, Xu X (2019). Long noncoding RNA MALAT1 correlates with cell viability and mobility by targeting miR223p in renal cell carcinoma via the PI3K/Akt pathway. Oncol Rep.

[271] Liu G, Zhao X, Zhou J (2018). LncRNA TP73-AS1 promotes cell proliferation and inhibits cell apoptosis in clear cell renal cell carcinoma through repressing KISS1 expression and inactivation of PI3K/Akt/mTOR signaling pathway. Cell Physiol Biochem.

[272] Su Y, Lu J, Chen X (2019). Long non-coding RNA HOTTIP affects renal cell carcinoma progression by regulating autophagy via the PI3K/Akt/Atg13 signaling pathway. J Cancer Res Clin Oncol.

[273] Lopez-Knowles E, Hernandez S, Malats N (2006). PIK3CA mutations are an early genetic alteration associated with FGFR3 mutations in superficial papillary bladder tumors. Cancer Res.

[274] Qian CN, Furge KA, Knol J (2009). Activation of the PI3K/AKT pathway induces urothelial carcinoma of the renal pelvis: identification in human tumors and confirmation in animal models. Cancer Res.

[275] Lv S, Wang W, Wang H (2019). PPARgamma activation serves as therapeutic strategy against bladder cancer via inhibiting PI3K-Akt signaling pathway. BMC Cancer.

[276] Lu JJ, Su YW, Wang CJ (2019). Semaphorin 4D promotes the proliferation and metastasis of bladder cancer by activating the PI3K/AKT pathway. Tumori.

[277] Gong Y, Qiu W, Ning X (2015). CCDC34 is up-regulated in bladder cancer and regulates bladder cancer cell proliferation, apoptosis and migration. Oncotarget.

[278] Fan Y, Song X, Du H (2014). Down-regulation of miR-29c in human bladder cancer and the inhibition of proliferation in T24 cell via PI3K-AKT pathway. Med Oncol.

[279] Noguchi S, Yasui Y, Iwasaki J (2013). Replacement treatment with microRNA-143 and -145 induces synergistic inhibition of the growth of human bladder cancer cells by regulating PI3K/Akt and MAPK signaling pathways. Cancer Lett.

[280] Li Y, Shan Z, Liu C (2017). MicroRNA-294 promotes cellular proliferation and motility through the PI3K/AKT and JAK/STAT pathways by upregulation of NRAS in bladder cancer. Biochemistry (Mosc).

[281] Zhai X, Xu W (2018). Long noncoding RNA atb promotes proliferation, migration, and invasion in bladder cancer by suppressing microRNA-126. Oncol Res.

[282] Li Z, Hong S, Liu Z (2018). LncRNA LINC00641 predicts prognosis and inhibits bladder cancer progression through miR-197-3p/KLF10/PTEN/PI3K/AKT cascade. Biochem Biophys Res Commun.

[283] Wang J, Ma W, Liu Y (2017). Long non-coding RNA HULC promotes bladder cancer cells proliferation but inhibits apoptosis via regulation of ZIC2 and PI3K/AKT signaling pathway. Cancer Biomark.

[284] Lv XY, Ma L, Chen JF (2018). Knockdown of DUXAP10 inhibits proliferation and promotes apoptosis in bladder cancer cells via PI3K/Akt/mTOR signaling pathway. Int J Oncol.

[285] Yang C, Li X, Wang Y (2012). Long non-coding RNA UCA1 regulated cell cycle distribution via CREB through PI3-K dependent pathway in bladder carcinoma cells. Gene.

[286] Hacioglu BM, Kodaz H, Erdogan B (2017). K-RAS and N-RAS mutations in testicular germ cell tumors. Bosn J Basic Med Sci.

[287] Feldman DR, Iyer G, Van Alstine L (2014). Presence of somatic mutations within PIK3CA, AKT, RAS, and FGFR3 but not BRAF in cisplatin-resistant germ cell tumors. Clin Cancer Res.

[288] Xu H, Feng Y, Jia Z (2017). AXIN1 protects against testicular germ cell tumors via the PI3K/AKT/mTOR signaling pathway. Oncol Lett.

[289] Gan Y, Wang Y, Tan Z (2016). TDRG1 regulates chemosensitivity of seminoma TCam-2 cells to cisplatin via PI3K/Akt/mTOR signaling pathway and mitochondria-mediated apoptotic pathway. Cancer Biol Ther.

[290] Wei J, Gan Y, Peng D (2018). Long non-coding RNA H19 promotes TDRG1 expression and cisplatin resistance by sequestering miRNA-106b-5p in seminoma. Cancer Med.

[291] Faiman B, Faiman M (2017). Living with hematologic cancer: recommendations, solutions. Cleve Clin J Med.

[292] Rajkumar SV (2016). Multiple myeloma: 2016 update on diagnosis, risk-stratification, and management. Am J Hematol.

[293] Ansell SM (2015). Hodgkin lymphoma: diagnosis and treatment. Mayo Clin Proc.

[294] Matsuki E, Younes A (2015). Lymphomagenesis in Hodgkin lymphoma. Semin Cancer Biol.

[295] Aravinth SP, Rajendran S, Li Y (2019). Epstein–Barr virus-encoded LMP1 induces ectopic CD137 expression on Hodgkin and Reed-Sternberg cells via the PI3K-AKT-mTOR pathway. Leuk Lymphoma.

[296] Desch AK, Hartung K, Botzen A (2020). Genotyping circulating tumor DNA of pediatric Hodgkin lymphoma. Leukemia.

[297] Zhu F, Guo H, Bates PD (2019). PRMT5 is upregulated by B-cell receptor signaling and forms a positive-feedback loop with PI3K/AKT in lymphoma cells. Leukemia.

[298] Dutton A, Reynolds GM, Dawson CW (2005). Constitutive activation of phosphatidyl-inositide 3 kinase contributes to the survival of Hodgkin's lymphoma cells through a mechanism involving Akt kinase and mTOR. J Pathol.

[299] Kuppers R, Engert A, Hansmann ML (2012). Hodgkin lymphoma. J Clin Invest.

[300] Georgakis GV, Li Y, Rassidakis GZ (2006). Inhibition of the phosphatidylinositol-3 kinase/Akt promotes G1 cell cycle arrest and apoptosis in Hodgkin lymphoma. Br J Haematol.

[301] Locatelli SL, Careddu G, Serio S (2019). Targeting cancer cells and tumor microenvironment in preclinical and clinical models of Hodgkin lymphoma using the dual PI3Kdelta/gamma inhibitor RP6530. Clin Cancer Res.

[302] Locatelli SL, Careddu G, Inghirami G (2016). The novel PI3K-delta inhibitor TGR-1202 enhances Brentuximab Vedotin-induced Hodgkin lymphoma cell death via mitotic arrest. Leukemia.

[303] Swerdlow SH, Campo E, Pileri SA (2016). The 2016 revision of the World Health Organization classification of lymphoid neoplasms. Blood.

[304] Armitage JO, Gascoyne RD, Lunning MA (2017). Non-Hodgkin lymphoma. Lancet.

[305] Crisci S, Di Francia R, Mele S (2019). Overview of targeted drugs for mature B-cell non-hodgkin lymphomas. Front Oncol.

[306] Sukswai N, Lyapichev K, Khoury JD (2020). Diffuse large B-cell lymphoma variants: an update. Pathology.

[307] Sorigue M, Sancho JM (2019). Recent landmark studies in follicular lymphoma. Blood Rev.

[308] Pfeifer M, Grau M, Lenze D (2013). PTEN loss defines a PI3K/AKT pathway-dependent germinal center subtype of diffuse large B-cell lymphoma. Proc Natl Acad Sci USA.

[309] Pasqualucci L, Khiabanian H, Fangazio M (2014). Genetics of follicular lymphoma transformation. Cell Rep.

[310] Freedman A (2018). Follicular lymphoma: 2018 update on diagnosis and management. Am J Hematol.

[311] Devan J, Janikova A, Mraz M (2018). New concepts in follicular lymphoma biology: from BCL2 to epigenetic regulators and non-coding RNAs. Semin Oncol.

[312] Pongas G, Cheson BD (2016). PI3K signaling pathway in normal B cells and indolent B-cell malignancies. Semin Oncol.

[313] Clayton E, Bardi G, Bell SE (2002). A crucial role for the p110delta subunit of phosphatidylinositol 3-kinase in B cell development and activation. J Exp Med.

[314] Okkenhaug K, Bilancio A, Farjot G (2002). Impaired B and T cell antigen receptor signaling in p110delta PI 3-kinase mutant mice. Science.

[315] Gopal AK, Kahl BS, de Vos S (2014). PI3Kdelta inhibition by idelalisib in patients with relapsed indolent lymphoma. N Engl J Med.

[316] Feng Y, Cu X, Xin M (2019). PI3Kdelta inhibitors for the treatment of cancer: a patent review (2015-present). Expert Opin Ther Pat.

[317] Patnaik A, Appleman LJ, Tolcher AW (2016). First-in-human phase I study of copanlisib (BAY 80–6946), an intravenous pan-class I phosphatidylinositol 3-kinase inhibitor, in patients with advanced solid tumors and non-Hodgkin's lymphomas. Ann Oncol.

[318] Markham A (2017). Copanlisib: first global approval. Drugs.

[319] Flinn IW, Miller CB, Ardeshna KM (2019). DYNAMO: a phase II study of duvelisib (IPI-145) in patients with refractory indolent non-hodgkin lymphoma. J Clin Oncol.

[320] Blair HA (2018). Duvelisib: first global approval. Drugs.

[321] Cui W, Ma M, Zheng S (2017). PIK3CA amplification and PTEN loss in diffused large B-cell lymphoma. Oncotarget.

[322] Chen L, Ouyang J, Wienand K (2019). CXCR4 upregulation is an indicator of sensitivity to B-cell receptor/PI3K blockade and a potential resistance mechanism in B-cell receptor-dependent diffuse large B-cell lymphomas. Haematologica.

[323] Bojarczuk K, Wienand K, Ryan JA (2019). Targeted inhibition of PI3Kalpha/delta is synergistic with BCL-2 blockade in genetically defined subtypes of DLBCL. Blood.

[324] Kataoka K, Nagata Y, Kitanaka A (2015). Integrated molecular analysis of adult T cell leukemia/lymphoma. Nat Genet.

[325] Seffens A, Herrera A, Tegla C (2019). STAT3 dysregulation in mature T and NK cell lymphomas. Cancers (Basel).

[326] Matutes E (2018). The 2017 WHO update on mature T- and natural killer (NK) cell neoplasms. Int J Lab Hematol.

[327] Fukuda R-I, Hayashi A, Utsunomiya A (2005). Alteration of phosphatidylinositol 3-kinase cascade in the multilobulated nuclear formation of adult T cell leukemia/lymphoma (ATLL). Proc Natl Acad Sci USA.

[328] Katsuya H, Cook LBM, Rowan AG (2018). Phosphatidylinositol 3-kinase-delta (PI3K-delta) is a potential therapeutic target in adult T-cell leukemia-lymphoma. Biomark Res.

[329] Ismail SI, Mahmoud IS, Msallam MM (2010). Hotspot mutations of PIK3CA and AKT1 genes are absent in multiple myeloma. Leuk Res.

[330] Hucthagowder V, Meyer R, Mullins C (2012). Resequencing analysis of the candidate tyrosine kinase and RAS pathway gene families in multiple myeloma. Cancer Genet.

[331] Frenquelli M, Caridi N, Antonini E (2020). The WNT receptor ROR2 drives the interaction of multiple myeloma cells with the microenvironment through AKT activation. Leukemia.

[332] Hofmann C, Stuhmer T, Schmiedl N (2014). PI3K-dependent multiple myeloma cell survival is mediated by the PIK3CA isoform. Br J Haematol.

[333] Ramakrishnan V, Kumar S (2018). PI3K/AKT/mTOR pathway in multiple myeloma: from basic biology to clinical promise. Leuk Lymphoma.

[334] Mimura N, Hideshima T, Shimomura T (2014). Selective and potent Akt inhibition triggers anti-myeloma activities and enhances fatal endoplasmic reticulum stress induced by proteasome inhibition. Cancer Res.

[335] Aronson LI, Davenport EL, Mirabella F (2013). Understanding the interplay between the proteasome pathway and autophagy in response to dual PI3K/mTOR inhibition in myeloma cells is essential for their effective clinical application. Leukemia.

[336] Richardson PG, Wolf J, Jakubowiak A (2011). Perifosine plus bortezomib and dexamethasone in patients with relapsed/refractory multiple myeloma previously treated with bortezomib: results of a multicenter phase I/II trial. J Clin Oncol.

[337] Jakubowiak AJ, Richardson PG, Zimmerman T (2012). Perifosine plus lenalidomide and dexamethasone in relapsed and relapsed/refractory multiple myeloma: a Phase I Multiple Myeloma Research Consortium study. Br J Haematol.

[338] Tolcher AW, Patnaik A, Papadopoulos KP (2015). Phase I study of the MEK inhibitor trametinib in combination with the AKT inhibitor afuresertib in patients with solid tumors and multiple myeloma. Cancer Chemother Pharmacol.

[339] Spencer A, Yoon SS, Harrison SJ (2014). The novel AKT inhibitor afuresertib shows favorable safety, pharmacokinetics, and clinical activity in multiple myeloma. Blood.

[340] Tasian SK, Teachey DT, Rheingold SR (2014). Targeting the PI3K/mTOR pathway in pediatric hematologic malignancies. Front Oncol.

[341] Valent P (2011). Targeting of leukemia-initiating cells to develop curative drug therapies: straightforward but nontrivial concept. Curr Cancer Drug Targets.

[342] Li J, Zhang J, Tang M (2016). Hematopoietic stem cell activity is regulated by pten phosphorylation through a niche-dependent mechanism. Stem Cells.

[343] Wu Y, Zhu H, Wu H (2019). PTEN in regulating hematopoiesis and leukemogenesis. Cold Spring Harb Perspect Med.

[344] Wu Y, Hu Y, Yu X (2019). TAL1 mediates imatinib-induced CML cell apoptosis via the PTEN/PI3K/AKT pathway. Biochem Biophys Res Commun.

[345] Roszak J, Smok-Pieniazek A, Stepnik M (2017). Transcriptomic analysis of the PI3K/Akt signaling pathway reveals the dual role of the c-Jun oncogene in cytotoxicity and the development of resistance in HL-60 leukemia cells in response to arsenic trioxide. Adv Clin Exp Med.

[346] Kosalai ST, Morsy MHA, Papakonstantinou N (2019). EZH2 upregulates the PI3K/AKT pathway through IGF1R and MYC in clinically aggressive chronic lymphocytic leukaemia. Epigenetics.

[347] Li L, Qi Y, Ma X (2018). TRIM22 knockdown suppresses chronic myeloid leukemia via inhibiting PI3K/Akt/mTOR signaling pathway. Cell Biol Int.

[348] Fuka G, Kantner HP, Grausenburger R (2012). Silencing of ETV6/RUNX1 abrogates PI3K/AKT/mTOR signaling and impairs reconstitution of leukemia in xenografts. Leukemia.

[349] Jiang MJ, Dai JJ, Gu DN (2017). MicroRNA-7 inhibits cell proliferation of chronic myeloid leukemia and sensitizes it to imatinib in vitro. Biochem Biophys Res Commun.

[350] Palacios F, Abreu C, Prieto D (2015). Activation of the PI3K/AKT pathway by microRNA-22 results in CLL B-cell proliferation. Leukemia.

[351] Yuan T, Yang Y, Chen J (2017). Regulation of PI3K signaling in T-cell acute lymphoblastic leukemia: a novel PTEN/Ikaros/miR-26b mechanism reveals a critical targetable role for PIK3CD. Leukemia.

[352] Wan L, Tian Y, Zhang R (2018). MicroRNA-103 confers the resistance to long-treatment of adriamycin to human leukemia cells by regulation of COP1. J Cell Biochem.

[353] Zhang Y, Zeng C, Lu S (2016). Identification of miR-125b targets involved in acute promyelocytic leukemia cell proliferation. Biochem Biophys Res Commun.

[354] Raffel S, Trumpp A (2016). miR-126 drives quiescence and self-renewal in leukemic stem cells. Cancer Cell.

[355] Zhang R, Tang P, Wang F (2019). Tumor suppressor miR-139-5p targets Tspan3 and regulates the progression of acute myeloid leukemia through the PI3K/Akt pathway. J Cell Biochem.

[356] Zhao L, Li Y, Song X (2016). Upregulation of miR-181c inhibits chemoresistance by targeting ST8SIA4 in chronic myelocytic leukemia. Oncotarget.

[357] Li Y, Gao L, Luo X (2013). Epigenetic silencing of microRNA-193a contributes to leukemogenesis in t(8;21) acute myeloid leukemia by activating the PTEN/PI3K signal pathway. Blood.

[358] Chen L, Jiang X, Chen H (2019). microRNA-628 inhibits the proliferation of acute myeloid leukemia cells by directly targeting IGF-1R. Onco Targets Ther.

[359] Zhao L, Shan Y, Liu B (2017). Functional screen analysis reveals miR-3142 as central regulator in chemoresistance and proliferation through activation of the PTEN-AKT pathway in CML. Cell Death Dis.

[360] Lu Y, Li Y, Chai X (2017). Long noncoding RNA HULC promotes cell proliferation by regulating PI3K/AKT signaling pathway in chronic myeloid leukemia. Gene.

[361] Sun MD, Zheng YQ, Wang LP (2018). Long noncoding RNA UCA1 promotes cell proliferation, migration and invasion of human leukemia cells via sponging miR-126. Eur Rev Med Pharmacol Sci.

[362] Yang Y, Dai W, Sun Y (2019). Long noncoding RNA linc00239 promotes malignant behaviors and chemoresistance against doxorubicin partially via activation of the PI3K/Akt/mTOR pathway in acute myeloid leukaemia cells. Oncol Rep.

[363] Ma L, Kuai WX, Sun XZ (2018). Long noncoding RNA LINC00265 predicts the prognosis of acute myeloid leukemia patients and functions as a promoter by activating PI3K-AKT pathway. Eur Rev Med Pharmacol Sci.

[364] Bertacchini J, Heidari N, Mediani L (2015). Targeting PI3K/AKT/mTOR network for treatment of leukemia. Cell Mol Life Sci.

[365] Brown JR (2016). The PI3K pathway: clinical inhibition in chronic lymphocytic leukemia. Semin Oncol.

[366] Kost SEF, Saleh A, Mejia EM (2019). Transcriptional modulation by idelalisib synergizes with bendamustine in chronic lymphocytic leukemia. Cancers (Basel).

[367] Bashash D, Delshad M, Safaroghli-Azar A (2017). Novel pan PI3K inhibitor-induced apoptosis in APL cells correlates with suppression of telomerase: an emerging mechanism of action of BKM120. Int J Biochem Cell Biol.

[368] Shah K, Moharram SA, Kazi JU (2018). Acute leukemia cells resistant to PI3K/mTOR inhibition display upregulation of P2RY14 expression. Clin Epigenetics.

[369] Perry JA, Kiezun A, Tonzi P (2014). Complementary genomic approaches highlight the PI3K/mTOR pathway as a common vulnerability in osteosarcoma. Proc Natl Acad Sci USA.

[370] Gupte A, Baker EK, Wan SS (2015). Systematic screening identifies dual PI3K and mTOR inhibition as a conserved therapeutic vulnerability in osteosarcoma. Clin Cancer Res.

[371] Gobin B, Battaglia S, Lanel R (2014). NVP-BEZ235, a dual PI3K/mTOR inhibitor, inhibits osteosarcoma cell proliferation and tumor development in vivo with an improved survival rate. Cancer Lett.

[372] Moore JBT, Loeb DM, Hong KU (2015). Epigenetic reprogramming and re-differentiation of a Ewing sarcoma cell line. Front Cell Dev Biol.

[373] Ren C, Ren T, Yang K (2016). Inhibition of SOX2 induces cell apoptosis and G1/S arrest in Ewing's sarcoma through the PI3K/Akt pathway. J Exp Clin Cancer Res.

[374] Zhao X, Fang Y, Wang X (2020). Knockdown of Ski decreases osteosarcoma cell proliferation and migration by suppressing the PI3K/Akt signaling pathway. Int J Oncol.

[375] Ye C, Yu X, Liu X (2018). miR-30d inhibits cell biological progression of Ewing's sarcoma by suppressing the MEK/ERK and PI3K/Akt pathways in vitro. Oncol Lett.

[376] Zhang S, Li D, Jiao GJ (2018). miR-185 suppresses progression of Ewing's sarcoma via inhibiting the PI3K/AKT and Wnt/beta-catenin pathways. Onco Targets Ther.

[377] Passacantilli I, Frisone P, De Paola E (2017). hnRNPM guides an alternative splicing program in response to inhibition of the PI3K/AKT/mTOR pathway in Ewing sarcoma cells. Nucleic Acids Res.

[378] Schadendorf D, Fisher DE, Garbe C (2015). Melanoma Nat Rev Dis Primers.

[379] Leonardi GC, Falzone L, Salemi R (2018). Cutaneous melanoma: from pathogenesis to therapy (review). Int J Oncol.

[380] Marsh Durban V, Deuker MM, Bosenberg MW (2013). Differential AKT dependency displayed by mouse models of BRAFV600E-initiated melanoma. J Clin Invest.

[381] Gibney GT, Smalley KS (2013). An unholy alliance: cooperation between BRAF and NF1 in melanoma development and BRAF inhibitor resistance. Cancer Discov.

[382] Nissan MH, Pratilas CA, Jones AM (2014). Loss of NF1 in cutaneous melanoma is associated with RAS activation and MEK dependence. Cancer Res.

[383] Deuker MM, Marsh Durban V, Phillips WA (2015). PI3'-kinase inhibition forestalls the onset of MEK1/2 inhibitor resistance in BRAF-mutated melanoma. Cancer Discov.

[384] Fernandez NB, Lorenzo D, Picco ME (2016). ROR1 contributes to melanoma cell growth and migration by regulating N-cadherin expression via the PI3K/Akt pathway. Mol Carcinog.

[385] Wang J, Li L, Liu S (2016). FOXC1 promotes melanoma by activating MST1R/PI3K/AKT. Oncotarget.

[386] Oliveira CS, de Bock CE, Molloy TJ (2014). Macrophage migration inhibitory factor engages PI3K/Akt signalling and is a prognostic factor in metastatic melanoma. BMC Cancer.

[387] Schlegel NC, von Planta A, Widmer DS (2015). PI3K signalling is required for a TGFbeta-induced epithelial–mesenchymal-like transition (EMT-like) in human melanoma cells. Exp Dermatol.

[388] Gao H, Liu R, Sun X (2019). STAT3-induced upregulation of lncRNA SNHG17 predicts a poor prognosis of melanoma and promotes cell proliferation and metastasis through regulating PI3K-AKT pathway. Eur Rev Med Pharmacol Sci.

[389] Yang Y, Zhang Z, Wu Z (2019). Downregulation of the expression of the lncRNA MIAT inhibits melanoma migration and invasion through the PI3K/AKT signaling pathway. Cancer Biomark.

[390] Chen X, Dong H, Liu S (2017). Long noncoding RNA MHENCR promotes melanoma progression via regulating miR-425/489-mediated PI3K-Akt pathway. Am J Transl Res.

[391] Wu J, Zhou MY, Yu XP (2019). Long noncoding RNA OR3A4 promotes the migration and invasion of melanoma through the PI3K/AKT signaling pathway. Eur Rev Med Pharmacol Sci.

[392] Liao Z, Zhao J, Yang Y (2018). Downregulation of lncRNA H19 inhibits the migration and invasion of melanoma cells by inactivating the NFkappaB and PI3K/Akt signaling pathways. Mol Med Rep.

[393] Dzneladze I, He R, Woolley JF (2015). INPP4B overexpression is associated with poor clinical outcome and therapy resistance in acute myeloid leukemia. Leukemia.

[394] Rijal S, Fleming S, Cummings N (2015). Inositol polyphosphate 4-phosphatase II (INPP4B) is associated with chemoresistance and poor outcome in AML. Blood.

[395] Gasser JA, Inuzuka H, Lau AW (2014). SGK3 mediates INPP4B-dependent PI3K signaling in breast cancer. Mol Cell.

[396] Fruman DA, Rommel C (2011). PI3Kdelta inhibitors in cancer: rationale and serendipity merge in the clinic. Cancer Discov.

[397] Macias-Perez IM, Flinn IW (2013). GS-1101: a delta-specific PI3K inhibitor in chronic lymphocytic leukemia. Curr Hematol Malig Rep.

[398] Chandarlapaty S, Sawai A, Scaltriti M (2011). AKT inhibition relieves feedback suppression of receptor tyrosine kinase expression and activity. Cancer Cell.

[399] Ghosh JC, Siegelin MD, Vaira V (2015). Adaptive mitochondrial reprogramming and resistance to PI3K therapy. J Natl Cancer Inst.

[400] Caino MC, Ghosh JC, Chae YC (2015). PI3K therapy reprograms mitochondrial trafficking to fuel tumor cell invasion. Proc Natl Acad Sci U S A.

